# Structural and biochemical analysis of family 92 carbohydrate-binding modules uncovers multivalent binding to β-glucans

**DOI:** 10.1038/s41467-024-47584-y

**Published:** 2024-04-23

**Authors:** Meng-Shu Hao, Scott Mazurkewich, He Li, Alma Kvammen, Srijani Saha, Salla Koskela, Annie R. Inman, Masahiro Nakajima, Nobukiyo Tanaka, Hiroyuki Nakai, Gisela Brändén, Vincent Bulone, Johan Larsbrink, Lauren S. McKee

**Affiliations:** 1 Division of Glycoscience, Department of Chemistry, KTH Royal Institute of Technology, AlbaNova University Centre, 106 91 Stockholm, Sweden; 2 Department of Life Sciences, Chalmers University of Technology, 41296 Gothenburg, Sweden; 3 Wallenberg Wood Science Center, Teknikringen 56-58, 10044 Stockholm, Sweden; 4 Department of Applied Biological Science, Faculty of Science and Technology, Tokyo University of Science, 2641 Yamazaki, Noda, Chiba, 278-8510 Japan; 5 Faculty of Agriculture, Niigata University, Niigata, 950-2181 Japan; 6 Department of Chemistry and Molecular Biology, University of Gothenburg, SE-405 30 Gothenburg, Sweden; 7 College of Medicine and Public Health, Flinders University, Bedford Park Campus, Sturt Road, SA, 5042 Australia; 8 Present Address: ZJU-Hangzhou Global Scientific and Technological Innovation Center, Zhejiang University, Hangzhou, 311215 China

**Keywords:** Polysaccharides, Soil microbiology, Water microbiology, X-ray crystallography, Biotechnology

## Abstract

Carbohydrate-binding modules (CBMs) are non-catalytic proteins found appended to carbohydrate-active enzymes. Soil and marine bacteria secrete such enzymes to scavenge nutrition, and they often use CBMs to improve reaction rates and retention of released sugars. Here we present a structural and functional analysis of the recently established CBM family 92. All proteins analysed bind preferentially to β−1,6-glucans. This contrasts with the diversity of predicted substrates among the enzymes attached to CBM92 domains. We present crystal structures for two proteins, and confirm by mutagenesis that tryptophan residues permit ligand binding at three distinct functional binding sites on each protein. Multivalent CBM families are uncommon, so the establishment and structural characterisation of CBM92 enriches the classification database and will facilitate functional prediction in future projects. We propose that CBM92 proteins may cross-link polysaccharides in nature, and might have use in novel strategies for enzyme immobilisation.

## Abstract (web summary)

Carbohydrate binding modules (CBMs) are non-catalytic domains found within multi-modular carbohydrate-active enzymes like glycoside hydrolases. Here, the authors show the crystal structures of two CBM family 92 members, which use three different surface binding sites to bind to β-glucans.

## Introduction

Carbohydrate-binding modules (CBMs) are low molecular weight, non-catalytic protein domains that bind carbohydrate ligands, and are classified by sequence homology into families on the carbohydrate-active enzymes (CAZy) database (www.cazy.org)^1^. At the time of writing, around 100 CBM families are described. In addition to family classification, CBMs can be categorised according to their ligand binding mode, determined by their protein structure, which conveys surface-binding, chain-binding, or small sugar-binding tendencies^2,3^.

Most often, CBMs are found within multi-modular CAZymes containing active domains such as glycoside hydrolases (GH), and their major role is thought to be the promotion of catalytic activity by facilitating or prolonging enzyme contact with substrate^2^. When a CBM binds to the same polysaccharide that an attached enzyme can hydrolyse, the reaction rate may increase^2^. In other situations, where the CBM binds to carbohydrates not targeted by the connected enzyme, catalytic conversion might be promoted by enzyme stabilisation^4^ or by tethering the enzyme to a complex substrate like an intact cell wall^5^. Multi-modularity is a particularly common feature of CAZymes in bacteria that rely on the secretion of extracellular enzymes for glycan foraging. These include the Bacteroidota (formerly Bacteroidetes) phylum, where several domains of unknown function (DUFs) associated with GHs remain uncharacterised.

The characterisation of DUFs from microbes with CAZyme-enriched genomes has led to the discovery of several novel GH and CBM families. Recently, the first member of CBM92 was described by Mei et al. ^6^. Domains in this family have previously been annotated as Bacterial Fascin-like Domains (BFLDs). They are found almost exclusively in bacteria, and have some structural similarity to the individual β-trefoil domains of the eukaryotic Fascin superfamily of actin-binding proteins (Pfam PF06268) mostly studied in vertebrates, particularly humans, and *Drosophila*^7–9^. The recently described founding member of CBM92 is a carrageenan-binding module^6^ appended to the κ-carrageenase enzyme Cgk16A, produced by the marine bacterium *Wenyingzhuangia aestuarii* OF219^10^. However, the first apparent demonstration in the literature of carbohydrate binding by a CBM92 protein appears to be the β−1,3-glucanase LamC from a myxobacterial *Corallococcus* species, where affinity gel electrophoresis showed that a BFLD at the N-terminus of a GH16 catalytic domain could bind to β−1,3-glucans^11^.

In this article, we present an extensive phylogenetic analysis of CBM92 sequences, which shows that most family members are attached to GHs with demonstrated or predicted activity on either chitin or diverse β-glucans. We have furthermore recombinantly produced and investigated 12 phylogenetically distinct CBM92 proteins found in soil and marine dwelling bacteria. The domains investigated bind with high specificity to linear β−1,6-glucans (i.e. pustulan^12^) and to polysaccharides with β−1,6-glucosidic branching points, such as laminarin and scleroglucan^13^. Linear β−1,6-glucans are found in the cell walls of some fungi^14–16^ and oomycetes^17–19^, and are easily extractable from lichenous fungi such as *Lasallia pustulata*^20^, while scleroglucan is produced by fermenting Sclerotium fungi^21^. Indeed, fungal biomass, and β−1,6-glucans in particular, are carbon sources strongly favoured by soil-dwelling Bacteroidota such as *Chitinophaga pinensis*^22,23^. As laminarin is abundant in the marine environment^24^, our analyses indicate that CBM92 domains are used by Bacteroidota in the recognition of important carbon sources in two significant ecosystems. To the best of our knowledge, CBM binding to β−1,6-glucans has largely only been found in CBM4 proteins^25,26^, so the definition of CBM92 with broadly conserved affinity for this linkage type significantly expands our view of the importance of pustulan.

**Fig. 1:**
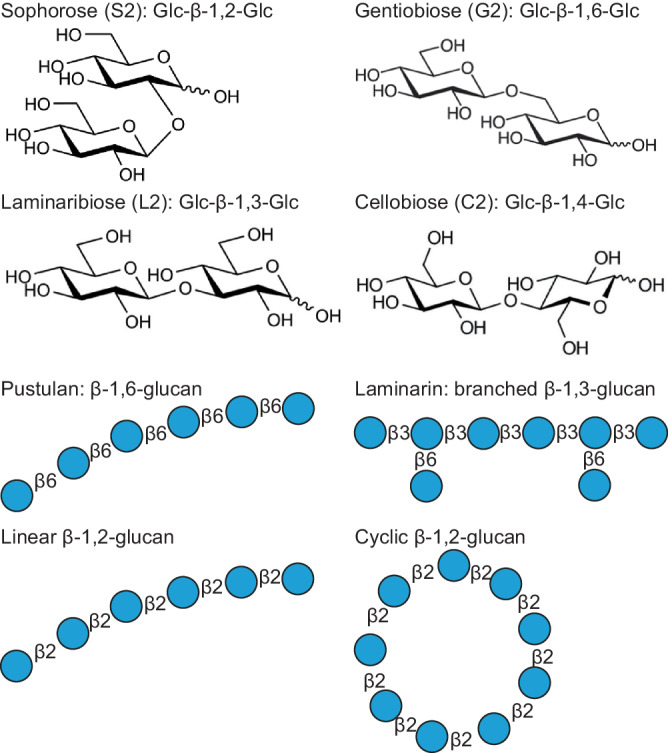
Structures of the main carbohydrates used in this investigation. In the cartoons, glucose is depicted as light blue circles. Although disaccharides have a high degree of conformational freedom, the different glucosidic linkages found in the ligands used in this study lead to significant spatial/structural differences in longer oligo- and polysaccharides. Scleroglucan has the same general structure as is depicted for laminarin, albeit with a far longer chain length and likely a higher degree of substitution. So-called yeast β-glucan extracted from the cell walls of *Saccharomyces* again has a similar structure, but with extended chains of β−1,6-linked glucosyl branching. Meanwhile, the twist arising from the β-1,2 linkage can produce cyclical polymers, and β−1,6-glucans can show a hook-like conformation^78,79^. Disaccharides are shown to represent each Glc-Glc linkage, but polysaccharides can be hundreds of Glc units in length.

In this work, we used complementary techniques to study the affinity and specificity of binding of CBM92 proteins to a series of glycans (Fig. 1) and uncovered an uncommon three-site mode of multivalent binding. We present crystal structures for two exemplary proteins cloned from the genome of *C. pinensis*, *Cp*CBM92A and *Cp*CBM92B, in complex with ligands. These structural data reveal three distinct carbohydrate-binding sites on the protein surface, with one site found within each of three structural subdomains (α, β, γ). We present a quantitative analysis of carbohydrate binding by several variant forms of *Cp*CBM92A, confirming that the binding abilities of all three sites are dependent on a conserved Trp residue^27^. The establishment and detailed characterisation of family CBM92 sheds light on the cell wall-targeting enzyme systems of environmental microbes, and will guide future microbial (meta)genome annotation studies. The predicted diversity in specificity of appended enzymes contrasts with the consistency of ligand preference we observe for the proteins studied here, and suggests that supporting the activity of an enzyme partner may not be the sole function of CBM92 domains.

## Results and discussion

### Family 92 carbohydrate-binding modules are commonly appended to glycoside hydrolases

**Fig. 2:**
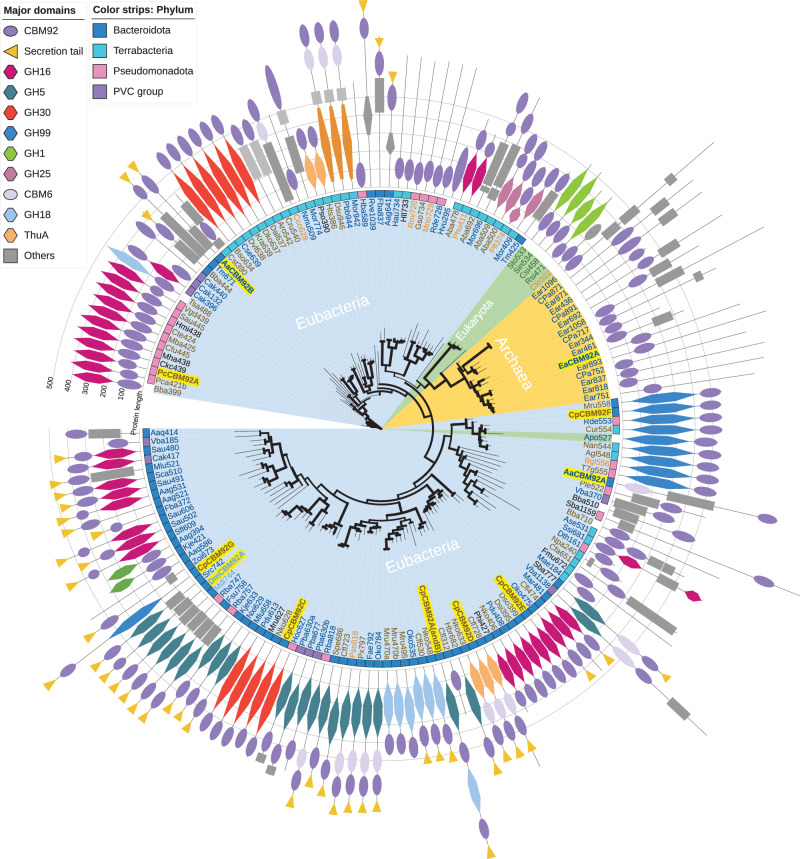
Phylogenetic depiction of the multi-modular proteins that contain CBM92 domains. Full protein sequences were aligned at the CBM92 domain, clustering proteins by domain architecture, and the phylogeny was analysed by maximum likelihood (iQtree web server, 1000 replicates). Bootstrap value is shown as branch thickness. Eubacteria, Eukaryota, and Archaea are respectively shaded with light blue, green, and yellow. Coloured squares on the outer ring indicate the phylum: Bacteroidota, Terrabacteria, Pseudomonadota, and PVC group are respectively shown in blue, light blue, pink, and purple. Pictograms depict the domains found in multi-modular proteins: see shape and colour key on the figure. Protein names contain abbreviated species names followed by the number of amino acids: see abbreviations and corresponding accession numbers in Supplementary Table 1. Protein names are respectively coloured brown or blue to indicate the host species is found in soil or water, where black means unknown. Light brown and light blue are soil or water environments with close association to plants.

The recent establishment of CBM92 as a family is supported by sequence comparison with other families. Indeed, in our phylogenetic analysis, family CBM92 forms a distinct clade with high bootstrap value (Supplementary Fig. 1). CBM92 domains are found in multi-modular proteins that in almost every case include at least one identifiable GH or polysaccharide lyase (PL) (Fig. 2). This indicates the possibility for CBM92 proteins to assist carbohydrate degrading activity by promoting enzyme contact with substrate. Indeed, the founding member of the family is a carrageenan-binding module appended to the κ-carrageenase enzyme Cgk16A^6^ produced by the marine bacterium *W. aestuarii*, a species that appears to be proficient at metabolising marine polysaccharides^10,28^. Carrageenan has structural features such as variable sulphation and anhydro-sugar moieties that are not found in terrestrial glycans^29^. Yet our preliminary phylogenetic investigations into CBM92 uncovered sequences from a broad range of non-aquatic microbes, suggesting that marine polysaccharides are not the only binding targets.

Using a CBM92 sequence from a soil bacterium as the search input, we identified 164 domains from 163 modular proteins as belonging to family CBM92, with non-redundant genus. Based on our analysis (Fig. 2), the family is mainly distributed among the Eubacteria, with some rare examples in Eukaryota and Archaea. Most CBM92-encoding species are found in soil, fresh water, and ocean ecosystems, including ocean sediment (Fig. 2). Among the Eubacteria, CBM92 is especially enriched in the phylum Bacteroidota, but can also be found among Pseudomonadota (formerly known as Proteobacteria), Terrabacteria, and in the PVC group (Planctomycetota, Verrucomicrobiota, and Chlamydiota). Approximately half of the CBM-containing multi-modular CAZyme proteins in our analyses are predicted to be secreted via the Bacteroidota-specific Type IX Secretion System (T9SS), as they possess the C-terminal domain that marks a protein for secretion via this pathway^30,31^, which has previously been highlighted as important for the secretion of polysaccharide-degrading enzymes^22,32^. A rare case of a eukaryotic CBM92-containing protein is a GH1 enzyme found in four Eudicot plant species, which carries the binding domain at its N-terminal end. The only animal genome that seems to encode a CBM92 domain is that of the wood-feeding termite *Coptotermes formosanus*. Indeed, both our analysis and a previous transcriptomic study^33^ suggest the occurrence of a protein in that species that contains a CBM92 and a CBM13 domain linked to a putative hemicellulose degrading enzyme.

Of note, the conserved ligand specificity we find for CBM92 proteins (discussed below) is in contrast to the apparent diversity in substrates targeted by the enzymes attached to these modules, which are predicted to include GH18 chitinases, GH16 β-1,3-glucanases and carrageenases, GH25 lysozymes, GH99 α-mannanases, and GH30 β−1,6-glucanases, as well as potentially highly diverse specificities from the multi-functional family GH5^34^. Generally, we see that the CBM92 domain is closely attached to its enzyme partner, to which it is connected via a short linker of less than 20 amino acids in most cases.

Sequences for CBM92 domains were extracted from full-length multi-modular protein sequences, and an independent evolutionary analysis was performed. The CBM92 domains are 125-150 amino acids long, and share an overall sequence identity of ≥ 37%. In the evolutionary tree of CBM92 (Supplementary Fig. 2), at least three distinct clades are seen, corresponding to the Eukaryota, Archaea, and Eubacteria, and within Eubacteria a distinct sub-clade of sequences derives from the Terrabacteria taxon. Since there are many Bacteroidota encoding one or more CBM92-containing protein(s), these likely entered Bacteroidota genomes at an early stage of evolution and then diverged. Conversely, few CBM92 domains occur in Pseudomonadota, and these do not form a distinct clade, which is inconsistent with the general evolutionary tree for these taxa^35^, and may indicate that for these species, CBM92 domains were acquired more recently via horizontal gene transfer.

### CBM92 proteins have three repeats defined as distinct subdomains, each with a conserved motif

Twelve CBM92 domains were selected for further analysis. Targets were chosen from species found in diverse habitats, while sampling sequence diversity from around the phylogenetic tree shown in Fig. 2. Furthermore, in their native multi-modular proteins, the selected domains are appended to GH enzymes from a number of different families (Fig. 2). Seven were chosen from the reasonably well-studied soil bacterium *C. pinensis*, which has one of the largest genomes and the highest number of CAZyme-encoding genes among Bacteroidota sequenced to date^1,22,36^. The *C. pinensis* domains analysed are appended to GH enzymes from families 5, 16, 18, and 99, which covers a broad range of potential enzyme substrates^37^. A further two domains were selected from the seawater-isolated *Aquimarina aggregata*^38^, both of which are appended to putative enzymes, with an additional CBM6 module in the full-length protein that contains *Aa*CBM92A. One CBM92 domain was selected from each of *Draconibacterium mangrovi* (isolated from river sediment in China^39^) and *Pyxidicoccus caerfyrddinensis* (isolated from soil in Caerfyrddin/Carmarthen in Wales^40^): *Dm*CBM92A is appended to GH5 and GH25 domains, while *Pc*CBM92A is attached to a GH16 domain. Finally, a CBM92 was selected from *Euryarchaeota archaeon* to explore the potential for functional binding in an archaeal representative.

**Fig. 3:**
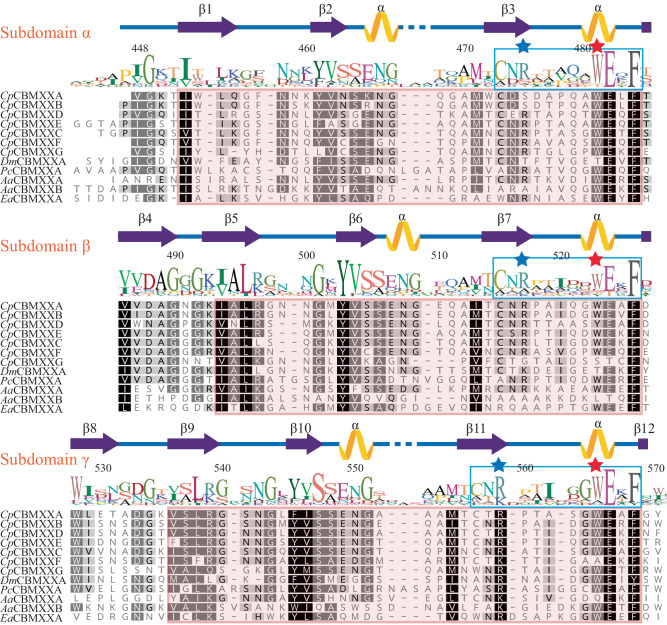
Sequence logo, secondary structure, and subdomains displayed on the alignment of twelve CBM92 domains. The pink shading on the alignment marks out sequences that are highly consistent across the larger dataset of 164 CBM92 sequences. The displayed amino acid numbers are based on the full-length sequence of the product of gene Cpin_2580, from which *Cp*CBM92A is derived. Three amino acid positions, W481, W523, and W565 (marked with pink stars within a highly conserved repeating WExF motif), were substituted with Ala to generate variants of *Cp*CBM92A for carbohydrate binding analysis. An Arg residue (blue stars) close to each WExF motif is proposed to contribute to binding. Full species names and accession numbers can be found in Supplementary Table 1.

From a sequence alignment of these 12 selected CBM92 domains, three repeat regions are observed and are named subdomains α, β, and γ (Fig. 3). The region of sequence highlighted in pink on Fig. 3 is conserved across all 164 CBM92 domains in our phylogeny. Secondary structure prediction suggests an enrichment in β sheets, indicating a β-trefoil structure, also found in e.g. CBM13^41^. A highly conserved ‘WExF’ sequence motif is present at the C-terminal end of each subdomain (Fig. 3). Interactions between carbohydrates and aromatic amino acids such as Trp are frequently important for CBMs^27,42^. We therefore speculated that the CBM92 proteins identified here have three binding sites each, centred around the three Trp residues of the ‘WExF’ motifs. A survey of other CBM92 proteins in our phylogeny show that the occurrence of three WExF motifs is widespread, although the Trp is lacking in one or more sites for some proteins (discussed below). Interestingly, the WExF motif is not found at all in the previously characterised carrageenan-binding protein^6^. Two Phe residues were suggested to be important for ligand binding in that protein, proposed to form a hydrophobic platform with support from a well-conserved Arg^6^. An alignment of the known and putative carrageenan-binders identified by Mei et al. with the proteins under analysis here shows that one of these Phe residues corresponds to the second WExF motif we find in almost all CBM92 proteins (Supplementary Fig. 3a, b). Our alignment further indicates that the carrageenan-binding proteins likely only have one binding site per protein, and that they represent a small sub-group within the family. These striking differences suggest that there are distinct modes of binding within the family, which warrants a further investigation of the binding specificities of CBM92.

### CBM92 domains bind to polysaccharides containing the Glc-β-1,6-Glc disaccharide unit

**Fig. 4:**
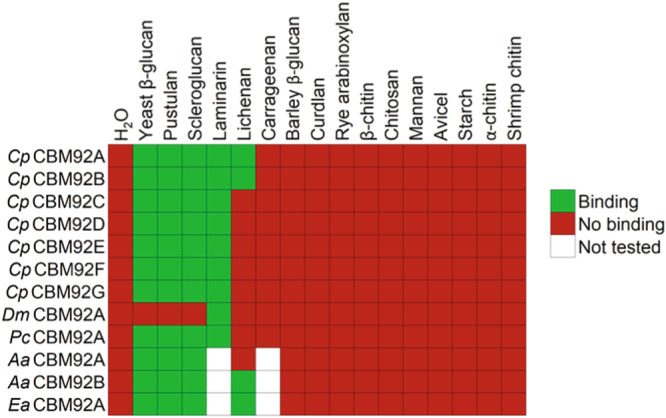
Qualitative binding determination of diverse CBM92 domains (left labels) to various polysaccharide ligands (top labels). For laminarin and carrageenan, binding was assayed by affinity gel electrophoresis. For all other ligands, a pull-down assay was used. The H_2_O samples contained no polysaccharide, as a control experiment. Each CBM domain was produced recombinantly without any other protein modules. The corresponding accession codes of the CBM domains shown in this figure can be found in Supplementary Table 1.

Gene segments encoding the 12 selected CBM92 domains were cloned and expressed as single-domain constructs in *E. coli* prior to purification. SDS-PAGE analysis confirmed successful production and purification for all recombinant domains (Supplementary Fig. 4). Carbohydrate binding was first investigated via pull-down assays and affinity gel electrophoresis using polysaccharides from diverse plant and microbial sources (see Materials and Methods for a full list of ligands tested). The heat map shown in Fig. 4 summarises the results of these binding assays, and the corresponding data can be found in Supplementary Fig. 5. The domains we tested show a consistent affinity for binding to polysaccharides containing the Glc-β-1,6-Glc linkage, namely pustulan (linear β-1,6-glucan), as well as laminarin, scleroglucan and yeast β-glucan (all consisting of β-1,3-glucan chains substituted with β-1,6-linked glucosyl residues). In some cases, there was some binding to lichenan, which comprises β-1,3- and β-1,4-linked glucosyl residues. Of note, *Dm*CBM92A, which naturally lacks two of the binding-site Trp residues we suggest are necessary for binding, did not noticeably bind to any of the tested polysaccharides except laminarin in this qualitative assay, although later experiments could measure some binding to yeast β-glucan (discussed below).

### Structural analysis reveals a β-trefoil fold with three carbohydrate binding sites

**Fig. 5:**
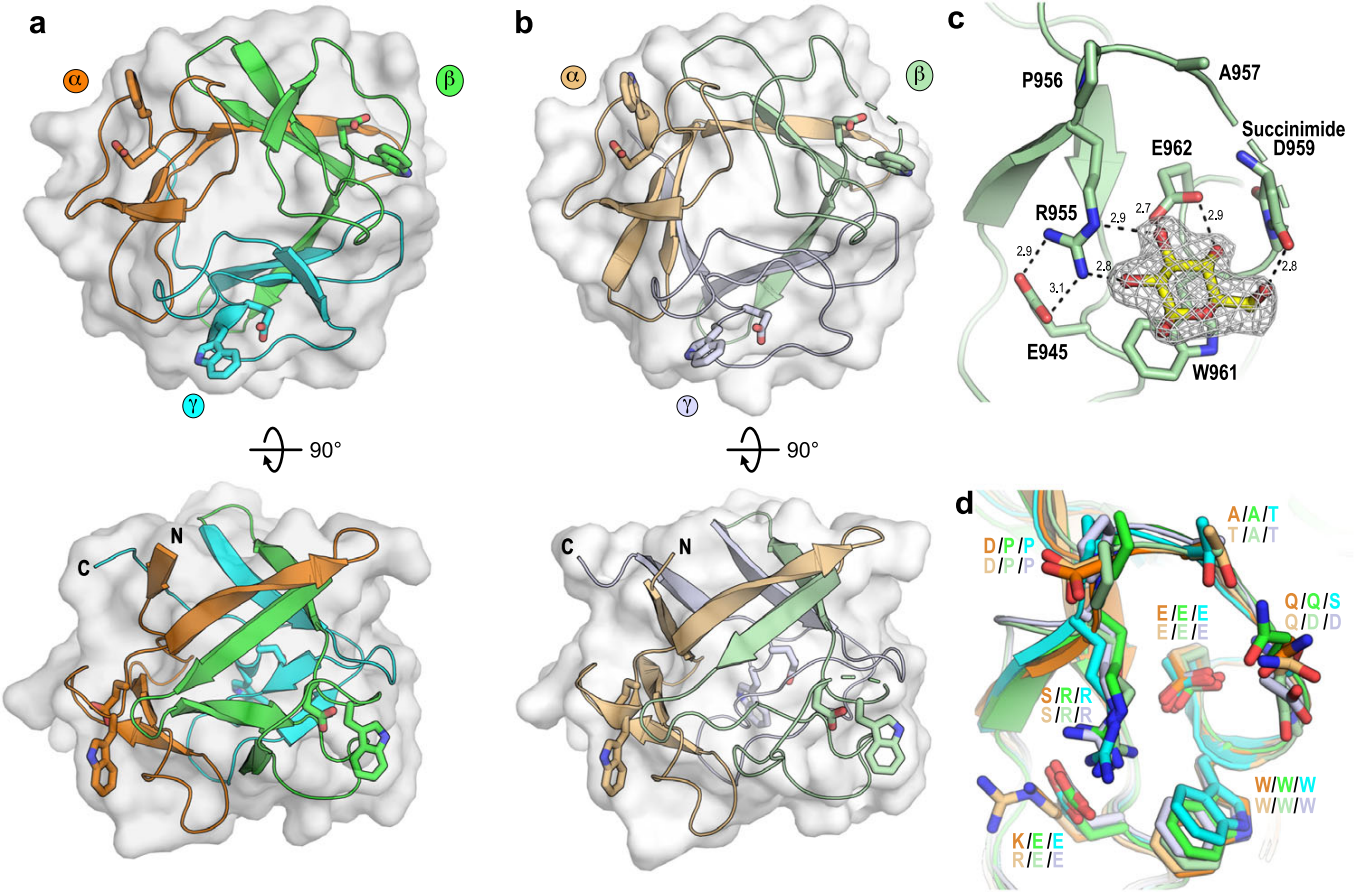
Structural analysis of two CBM92 domains reveals three subdomains and three potential ligand binding sites. Overall structures of (**a**) *Cp*CBM92A and (**b**) *Cp*CBM92B with their subdomains distinctly coloured and their ligand binding Trp and Glu residues shown as sticks. **c** The β-subdomain of *Cp*CBM92B in complex with glucose. Hydrogen bond distances are shown and the density from the 2Fo-Fc electron density map carved 1.6 Å around the glucosyl ligand and contoured at 1.0σ. **d** Overlay of the *Cp*CBM92A and B subdomains showing sequence conservation within all putative binding sites. Single letter residue codes are coloured based on the subdomains shown in panels **a** and **b**, and are labelled for subdomains α/β/γ, in that order, with the *Cp*CBM92A codes shown above those for *Cp*CBM92B.

To probe the mode of binding of CBM92 domains, we successfully determined the protein structures of the *C. pinensis* proteins *Cp*CBM92A and *Cp*CBM92B by macromolecular crystallography. As was predicted by sequence analysis, both proteins form a β-trefoil structure comprised of 12 β-strands arranged into 3 subdomains (α, β, and γ), similar to β-trefoil domains found in Fascin and CBM13 proteins^9,41^ (Fig. 5a, b). Soaking experiments of the *Cp*CBM92B protein crystals with glucose, gentiobiose (G2: Glc-β-1,6-Glc), and sophorose (S2: Glc-β−1,2-Glc) revealed a binding cleft within each subdomain comprising a Trp-Glu binding motif, again implying three polysaccharide binding sites per protein (Fig. 5c). Adding either G2 or S2 to the protein crystals led to binding of the non-reducing end sugar in the binding cleft. The electron density for the reducing end sugar was observable but difficult to model accurately, although it notably projected away from the protein (Supplementary Fig. 6). This suggests the capacity for end-on binding to glucose monosaccharides and glucan oligo/polysaccharides of potentially any linkage type. In each ligand complex, the glucosyl unit stacks with the conserved Trp with the O3 and O4 of the sugar positioned by hydrogen bonding with the Oε1 and Oε2 of the conserved Glu. In the binding site of *Cp*CBM92B subdomain β, the protein is observed to further interact with the glucosyl unit through the guanidine group of Arg955 with the O2 of the sugar, and through the carbonyl of a succinimide formed in place of Asp959 with the sugar O6 (Fig. 5c and Supplementary Fig. 7). Succinimide can form as a result of cyclising dehydration from nucleophilic attack of the main-chain N atom on the γ-carbon of Asn and Asp side chains^43,44^, and is rarely seen in protein structures. Indeed, only 45 protein entries containing this chemical group are currently reported in the PDB^45^. In our investigation it was found only in the β-subdomain of *Cp*CBM92B and it may be an artefact of protein production or crystallisation. Collectively, the binding modes observed with the ligand complexes reveal the possibility for extensions from both the O1 and O6, presumably enabling binding along a β−1,6-glucan chain such as in pustulan, and additionally binding to β-1,6-linked glucosyl substitutions in, for example, scleroglucan or laminarin. The binding cleft Arg residue in the β-subdomain of *Cp*CBM92B is found in subdomains β and γ in both *Cp*CBM92A and -B, but is substituted with a Ser in the binding clefts of subdomain α in both proteins (Fig. 5d). This substitution in the α site leads to a substantial increase in accessibility around the glucosyl unit’s O2, which may permit binding to oligo- or polysaccharide extensions from this position. In the paper by Mei et al. describing Cgk16A, the founding member of family CBM92, the authors propose that a conserved Arg may be responsible for interacting with the sulphate groups of that protein’s carrageenan ligand^6^, but our data indicate that it contributes to binding to non-sulphated glycan ligands as well (Supplementary Figs. 3 and 6).

### Structural comparison with homologues

*Cp*CBM92A and *Cp*CBM92B share structural similarity with β-trefoil proteins from CBM13, a multivalent family that includes single-domain galactose- or mannose-binding plant lectins as well as CBM domains found within larger CAZymes. Structural homologues to our CBM92 domains include the ricin B-like agglutinin domain from *Marasmius oreades*^46^, an arabinose-binding CBM domain in a GH27 β-l-arabinopyranosidase from *Streptomyces avermitilis*^47^, the CBM domain in CEL-III from *Cucumaria echinate*^48^, the xylose/xylan-binding CBM domain in the xylanase Xyn10A from *Streptomyces olivaceoviridis* E-86^49^, and actinohivin from *Longispora albida* K97-0003T^50^. Structural alignment with these proteins yields Cα root mean square deviation values of 1.5 to 2.5 Å despite low (8-20%) sequence identity. The ligand binding regions in CBM13 are also found in similar surface exposed clefts, with each protein containing three equivalent clefts as part of the trefoil fold. All of these proteins use an aromatic residue and an acidic residue to mediate ligand binding. However, the families differ in the origin of those residues, which ultimately leads to substantially different ligand binding modes (Supplementary Fig. 8). For example, the ricin B-like agglutinin domain from *M. oreades*, the CBM domain in β-l-arabinopyranosidase from *S. avermitilis*, the CBM domain in CEL-III from *C. echinate*, and the CBM domain in Xyn10A from *S. olivaceoviridis* E-86 all contain acidic residues originating from β2 and aromatic residues originating from β3 of the subdomains, effectively shifting the principal binding site by more than 5 Å compared to *Cp*CBM92A and *Cp*CBM92B. Other CBM13 members, such as actinohivin from *L. albida* K97-0003T, also use an acidic residue from β2 but their aromatic residues reside on a loop, or small helical section, preceding β4 of the subdomain. In CBM92, the aromatic residue originates from the loop preceding β4 but distinctly has the acidic residue also originating from this loop, leading to the principal binding site being perpendicular to that observed in CBM13 members such as actinohivin. Collectively, while all the proteins comprise a similar overall fold and use similar residues to mediate binding, the location of the residues leads to distinct ligand binding modes.

### Exploring the functionality and ligand specificity of three putative binding sites in CBM92

The crystal structures with glucose-based ligands provide evidence for chain-end binding to the non-reducing end of a ligand, with space for potential extension at O2 and O6, which would additionally permit mid-chain binding to glycans with those linkages. According to the crystal structures, mid-chain binding to e.g. β−1,3-glucan or β-1,4-glucan would not be possible. This matches our observations from the qualitative polysaccharide binding assays described above, which suggested some linkage-based selectivity in ligand binding. We used isothermal titration calorimetry (ITC) to explore the binding affinities of *Cp*CBM92A to glucose and glucose-based disaccharides. We were able to determine binding parameters for glucose, G2, and S2, while binding to C2 and L2 could not be reliably measured due to low signal and non-saturating isotherms. These experiments showed stronger binding to G2 and S2 than to glucose, perhaps reflecting the dual potential orientations of the longer ligands in the binding sites. Table 1 shows the parameters of binding determined for *Cp*CBM92A, and the corresponding data can be found in Supplementary Fig. 9.

**Table 1:** Binding parameters of the interactions between *Cp*CBM92A and three ligands as determined by ITC analysis

Ligand	*N*	K_D_	ΔH (kcal/mol)	ΔG (kcal/mol)	−TΔS (kcal/mol)
**Glucose**	3	1.84 ± 0.2 mM	−1.98 ± 0.152	−3.73	−1.75
**Gentiobiose**	3	202 ± 40 µM	−1.57 ± 0.1	−5.04	−3.47
**Sophorose**	3	882 ± 107 µM	−1.84 ± 0.14	−4.17	−2.33

Data represent the binding affinity of a whole protein with three functional binding sites. Data were fit using the Origin software, applying a model with three equivalent binding sites (*N* = 3, single-site binding).

To probe the respective functions of the three putative glycan binding sites, a series of modified constructs were generated for *Cp*CBM92A, systematically altering the Trp in each WExF motif. Variants with single (W481A α site, W523A β site, W565A γ site variants), double (W481A/W565A, W481/W523A, W523A/W565A), and triple (W481A/W523A/W565A) binding site substitutions were produced using site-directed mutagenesis (red stars in Fig. 3 show the positions of the residues modified). The doubly substituted W481/W523A variant showed no protein production despite optimisation attempts, while the W481A/W565A form proved to be highly unstable during protein production; as a result, these versions of the protein could unfortunately not be purified or characterised. The melting points of *Cp*CBM92A and all successfully produced variants were investigated, and suggested that protein structure was intact in the modified forms, which all showed similar melting point profiles (Supplementary Fig. 4). Pull-down assays revealed that the single mutation variants showed the same binding specificities as the wild-type, while the double and triple variants showed impaired or abolished binding (Supplementary Fig. 5a), confirming that there are no further unrecognised binding sites in the protein.

**Fig. 6:**
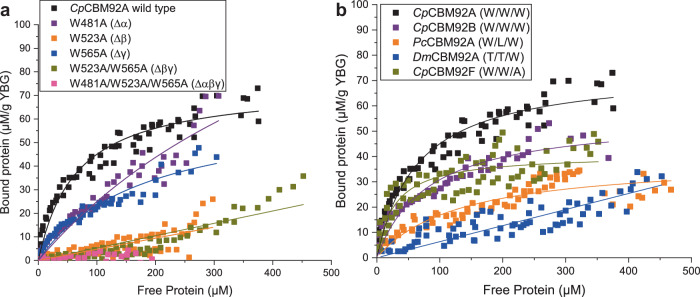
Depletion isotherms of CBM92 domains binding to the insoluble polysaccharide yeast β-glucan. **a** Binding site variants of *Cp*CBM92A were generated, wherein a key Trp residue was converted to Ala in one or more binding sites, as indicated. Binding data for the wild type and variant forms are presented. **b** Depletion isotherms are compared for several wild type CBM92 domains that differ in the presence or absence of a Trp in the α/β/γ binding site, as indicated by the X/X/X nomenclature. Full species names and accession numbers can be found in Supplementary Table 1.

Due to weak binding, satisfactory ITC experiments could not be performed for the variant forms of *Cp*CBM92A. Instead, a series of depletion isotherms were performed using the ligand yeast β-glucan, which comprises a backbone of β-1,3-glucan with regular extended sidechains of β−1,6-linked glucosyl units. Binding curves could not be saturated due to protein precipitation at high concentrations, so accurate K_D_ values could not be deduced from these data. However, lines of best fit determined using a Langmuir isotherm fitting model are shown to allow a qualitative comparison of binding strengths (Fig. 6). The wild type and all variant forms of *Cp*CBM92A were first assessed, to investigate the relative contribution to binding made by each site (Fig. 6a). The loss of the Trp residue from either the β or γ binding site (W523A and W565A variants, respectively) caused a major shift in apparent binding ability, with the loss of the β site having the most profound effect. This indicates that for *Cp*CBM92A, the β site likely has the strongest affinity for the ligand. We also see that the α site knockout shows only a small loss of binding ability compared to the wild type, but that there is some residual binding in the β/γ site variant W523A/W565A, suggesting that the wild type α site does make some small contribution to binding in the full protein. The α binding site of *Cp*CBM92A differs from the other two in that it lacks an otherwise well-conserved adjacent Arg (Fig. 3) that likely supports binding by interacting with a glucose ligand and by creating a topographic ‘wall’ for the binding site (Supplementary Fig. 5b).

Overall, the depletion isotherm data for variant forms of *Cp*CBM92A indicate that a greater number of functional (i.e. Trp-containing) binding sites leads to stronger overall binding to the polysaccharide yeast β-glucan. From these data it is not possible to determine whether this results from merely additive or truly avid binding. As there is some natural variety within CBM92 in the number of Trp-containing binding sites within wild type proteins (Fig. 3), we were motivated to perform depletion isotherms for a series of native proteins with differing binding site sequences (Fig. 6b). We see the weakest binding from *Dm*CBM92F, which only has Trp in the γ site, and gave an isotherm highly similar to that obtained for the β/γ variant W523A/W565A of *Cp*CBM92A. For *Cp*CBM92F and *Aa*CBM92B, which both lack one functional site, binding is compromised compared to wild type *Cp*CBM92A and *Cp*CBM92B, which both have three binding site Trp residues. In short, these data agree with observations from the *Cp*CBM92A variants and show that more Trp-containing binding sites leads to stronger interactions with ligand.

Finally, the label-free technique bio-layer interferometry (BLI) was employed, as this method has proven useful in measuring multivalent carbohydrate–protein interactions^51,52^. BLI works best with relatively high molecular weight ligands, although these must be soluble. Previous BLI experiments on carbohydrate-protein interactions mainly used streptavidin sensors^53^ and biotinylated Fab-conjugated glycans^53,54^. In this study, we instead used Ni-NTA sensors, wherein the sensor binds to the His_6_ tag on recombinant proteins. The interferometry variation during ligand association/dissociation steps were analysed in real-time.

Binding to sophoropentaose (S5), laminarin, and scleroglucan was studied using BLI for *Cp*CBM92A and its variants (Supplementary Fig. 10). Using the S5 ligand at a concentration of 10 µM enabled K_D_ values to be determined, as presented in Table 2. The α and γ site variants (respectively the W481A and W565A forms) show a binding profile that is highly similar to that of the wild type *Cp*CBM92A, indicating that the contributions of those sites to overall affinity is very minor. Conversely, the W523A β site variant shows a non-detectable degree of binding to S5, again confirming that this is the strongest binding site on the protein and that it may be particularly critical with certain ligands. The polysaccharides laminarin and scleroglucan are heterogeneous and polydisperse, so molar concentrations cannot be accurately measured. As a result, K_D_ values could not be determined for these interactions using BLI (Supplementary Fig. 10). Nonetheless, the general trend in these data echoes that from the depletion isotherm experiments, with stronger binding interactions again correlating with a greater number of intact Trp binding sites (Supplementary Fig. 10). A response value from BLI is measured as a nm shift in the interference pattern and is proportional to the number of molecules bound to the surface of the biosensor. Comparing the maximum response values obtained with laminarin as the ligand indicates that the wild type, α site variant, and γ site variant forms of *Cp*CBM92A saturate at roughly the same ligand concentrations, indicating highly similar binding affinities. By contrast, the β site variant reaches saturation more slowly in terms of ligand concentration, consistent with reduced binding affinity. With scleroglucan as ligand, which could be tested at higher concentrations than sophorose, there is a clear loss of binding in the W565A γ site variant, whereas loss of the α site (W481A) exerts a minimal effect on binding. In the doubly substituted variant where only subdomain α is unchanged from wild type, the binding profile is close to that of the triple variant, showing no binding to laminarin or scleroglucan. Overall, the BLI data re-confirm that the β site is contributing the most to CBM affinity for ligand, and indicate that the γ and α sites make lesser contributions to overall binding. Native PAGE analysis of binding to laminarin also indicated that the β binding site is the strongest, as the W523A β site variant showed the greatest reduction in mobility retardation, while the mobility of the W481A and W565A variants more closely resembles that of the wild type protein (Supplementary Fig. 5b). Although the BLI and depletion isotherm studies presented here show that there is some loss of overall binding capacity when the α or γ site Trp is lost, the affinity of these sites for ligand is likely to be comparatively low.

**Table 2:** Kinetic parameters of the interaction between *Cp*CBM92A variants and S5

*Cp*CBM92A variants	K_D_ (µM)	K_on_ (M^−^^1^s^−^^1^)	K_off_ (s^−^^1^)
**wild type**	6.43 ± 0.05	210.4 ± 1.6	1.35E-03 ± 3.32E-06
**W481A (Δα)**	4.56 ± 0.03	205.1 ± 1.08	9.36E-04 ± 2.14E-06
**W523A (Δβ)**	ND	ND	ND
**W565A (Δγ)**	7.19 ± 0.04	161.8 ± 0.96	1.16E-03 ± 2.05E-06
**W523A/W565A (Δβγ)**	ND	ND	ND
**Δαβγ**	ND	ND	ND

CpCBM92A variants were pre-immobilised on BLI NiNTA sensor, and S5 as ligand. The concentration of S5 was 10 µM. The measurement is based on 1:1 fitting model analysed in software (Octet Software Version 10.0). The binding and fitting curves (1:1 model) are shown in Suppl. Fig. 10. ND denotes no binding was detected.

### Implications of CBM92 binding to β−1,6-glucan

By characterising 12 examples, we have shown that CBM92 domains from distinct microbial species are capable of binding to glucose, gluco-oligosaccharides with β-1,2- or β−1,6- linkages, and to long chain glucans containing β−1,6-linked glucose moieties (pustulan, scleroglucan, yeast β-glucan, and laminarin). Previously characterised examples of CBM92-containing proteins bound to β−1,3-glucan^11^ and carrageenan^6^: both of those domains bind to the same polysaccharide as their appended enzymes can target, suggesting a likely role in enzyme potentiation^2^. Indeed, our phylogenetic analyses show that a number of CBM92 domains are attached to predicted β−1,6-glucanases from enzyme family GH30 (sub-family 3)^55^, and these may be expected to show the same kind of rate potentiation. The natural substrate for these enzymes may be polymeric pustulan as found in lichenous fungi^20^ or it may be shorter chains of β-1,6-glucan such as can be found in the cell walls of certain oomycetes^18^. However, the β-1,6-glucan-binding CBM92 domains characterised in this work are appended to CAZymes with a range of different predicted activities, suggesting that not every member of the family is involved in direct binding to the substrate of an enzyme partner. As β-1,6-glucosidic linkages are found in the cell walls and secretions of marine plants and soil fungi, it may be that tethering, for example, a chitinase^56^ or β−1,3-glucanase to a complex cell wall substrate matrix does have a rate-enhancing proximity effect in natural systems^5^.

**Fig. 7:**
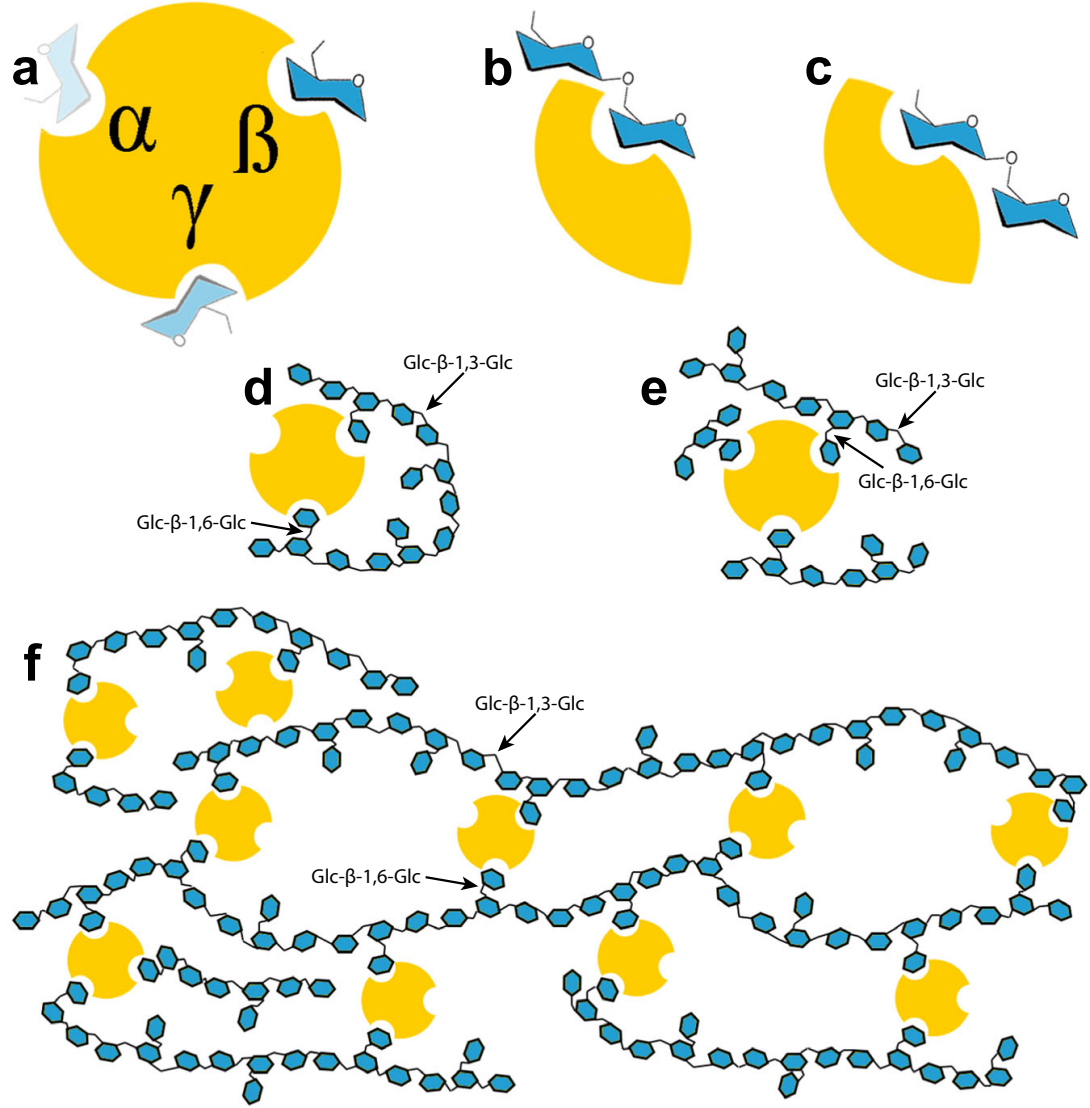
Theoretical model of *Cp*CBM92A binding to diverse β-glucans. **a** The wild type protein has three Trp-containing binding sites, depicted with a residue of glucose ligand within each. The more intensely the ligand is coloured, the higher the affinity to the depicted binding site. **b**, **c** The Glc-β-1,6-Glc disaccharide gentiobiose can bind in two potential orientations, with either the reducing-end or the non-reducing end sugar in the binding site. **d**–**e** *Cp*CBM92A binds to laminarin, a β−1,3-glucan with single sugar β-1,6-Glc decorations. Certain chains of laminarin likely have the flexibility for more than one substitution per chain to interact with the protein. **f** A favoured ligand for *Cp*CBM92A is scleroglucan, a very long chain and high molecular weight polysaccharide with a molecular structure similar to that of laminarin. Scleroglucan chains are not likely to be as flexible as laminarin-oligosaccharides, but a protein-polysaccharide network is speculated to form with long chains of this ligand, inter-locked by *Cp*CBM92A. Examples of Glc-β−1,3-Glc and Glc-β−1,6-Glc linkages are indicated with arrows in panels **d**–**f**.

In addition, the potential multivalent nature of CBM92 glycan binding might be significant, as it could lead to the formation of protein-polysaccharide networks that may stabilise enzymes in a manner conceptually similar to the use of immobilisation in industry. In a study characterising a CBM6 protein with two binding sites showing different modes of interaction with the β-1,3-glucan backbone of laminarin, Jam et al. proposed a model for CBM-mediated cross-linking of oligolaminarin chains up to 12 glucosyl units in length^57^. The three binding sites of CBM92, which our data suggest all make some contribution to overall binding, may permit a similar cross-linking of ligands in soil and water environments. The biological implications of this remain unclear, but from a biotechnological perspective, it may suggest that CBM92 domains have use as fusion tags for immobilisation of recombinant proteins on polysaccharide surfaces. Pustulan in particular is a strong candidate for an immobilisation surface, as it is inert and insoluble, and easily recoverable from water by centrifugation or filtration. Additional experiments are needed to determine whether this cross-linking interaction is occurring and if it has a stabilising effect on appended enzymes. In Fig. 7 we depict hypothetical models for how *Cp*CBM92A might interact with the various ligands analysed in this study. The model depicts two potential binding orientations for gentiobiose. If a longer oligosaccharide ligand, such as moderate chain length laminarin, were flexible enough, it may be able to sit in multiple binding sites on one protein, an interaction previously proposed for the bivalent CBM6 protein studied by Jam et al.^57^. A similar phenomenon may be feasible with sophoropentaose, which might be long enough to reach two binding sites on protein. In addition, with a very long chain ligand such as scleroglucan, a cross-linked protein-polysaccharide network may form if multiple binding sites of one protein interact with different ligand chains.

## Methods

### Carbohydrates utilised

Most polysaccharides used in protein binding assays were obtained from commercial suppliers. Chitosan, α-chitin, and β-chitin were obtained from Maharani Chitosan PTV, Ltd. (Gujarat, India), while scleroglucan and pustulan were purchased from Carbosynth, UK. Microcrystalline cellulose (Avicel), starch, laminarin, and birchwood xylan were from Sigma Aldrich, Germany. Barley β-glucan, oat spelt xylan, ivory nut mannan, curdlan, and lichenan were purchased from Megazyme, Ireland. Glucose was purchased from Sigma-Aldrich. The disaccharides cellobiose, gentiobiose, laminaribiose, and sophorose were all purchased from Megazyme. Sophoropentaose and linear β-1,2-glucans were prepared in-house using 1,2-β-oligoglucan phosphorylase and β-1,2-glucanase as described previously^58–60^. The weight and number averaged molecular weights of linear β-1,2-glucans are 7600 and 6200, respectively. The cyclic β-1,2-glucans were a gift from Mie University, Japan and comprised 17–24 glucose units.

### Bioinformatics

Database searches for CBM92 homologues were carried out using BLASTP with *C. pinensis Cp*CBM92A protein as query against the non-redundant protein sequences dataset of the Genbank database at the National Center for Biotechnology Information (NCBI) (http://www.ncbi.nlm.nih.gov). Sequences containing homologues to *Cp*CBM92A were selected to generate a CBM92-containing-protein subset for further analysis. This subset was evaluated using the taxonomy browser at NCBI. Incomplete and redundant entries were removed. Additionally, only one exemplary species was selected from each genus, and the final dataset contained sequences of 163 modular proteins. Supplementary Table 1 gives details on all proteins used in the bioinformatic analyses.

Protein sequence alignments were created using the Clustal Omega (for full-length protein) or Clustal Muscle (CBM92 domains only) tools from the European Molecular Biology Laboratory (EMBL) (https://www.ebi.ac.uk/Tools/msa). Sequence logos were generated from alignments using the GENEIOUS software (www.geneious.com). Alignments were applied to maximum likelihood analysis using IQtree with a bootstrap value of 1000, and with the substitution model VT + F + G4 automatically identified^61^. The tree output from IQtree was visualised using the Interactive Tree Of Life (iTOL) tool (https://itol.embl.de/). The final phylogenetic tree for full-length proteins was rooted by a clade of 10 Proteobacterial sequences, and the tree for CBM92 domains only was rooted by a clade of 4 Eukaryotic sequences. The CBM92 evolutionary tree was provisionally examined as a circular phylogeny with different taxa as root, e.g., Eukaryota, Archaea, Terrabacteria, and also as an unrooted tree and we could not find strong evidence of any obvious root taxon.

The 12 proteins that were selected for biochemical analysis in this paper were further analysed by protein sequence alignments to illustrate secondary structural elements of the CBM92 family. 6 of the 12 proteins were used for a broader phylogenetic analysis, comparing against other known CBM families using the same methods as described above. This analysis used 1-3 sequences selected from each CBM family in CAZy.

### Gene cloning and mutagenesis

Certain genes explored in this study were synthesised in a proprietary vector by ThermoFisher GeneArt; these were then sub-cloned into the expression vector pET21a (ThermoFisher), which carries a C-terminal His_6_-tag and confers ampicillin resistance. Other genes were cloned in-house from genomic *C. pinensis* DNA (DSMZ, Germany). See Supplementary Table 2 for details on the cloning strategy used to generate each construct. Snapgene version 5.3 was used for design of primers for genes cloned in-house from genomic DNA. Point mutations of *Cp*CBM92A were generated using site-directed mutagenesis of the *Cp*CBM92A construct; see Supplementary Table 3 for the sequences of primers utilised.

### Production and purification of recombinant proteins

Plasmids containing the genes of interest were transformed into *E. coli* BL21 (λDE3) (Life Technologies) by heat shock at 42 °C for 30 s. Cells were grown at 37 °C with shaking in selective LB medium containing ampicillin (50 µg mL^−^^1^) for 2–3 h until OD_600_ reached 0.5. At this point, gene expression was induced by the addition of 0.2 mM IPTG (isopropyl-d-galactopyranoside) and the temperature was lowered to 16–20 °C. Protein production proceeded for ~16 h. Cells were then collected by centrifugation at 6000 x *g* for 10 min. Cells were resuspended in TALON buffer A (50 mM sodium phosphate pH 6.5 or 7.4 with 300 mM sodium chloride) and lysed by sonication, followed by centrifugation at 35,000 x *g* for 30 min. The pH value of buffer A had to be optimised for certain proteins as their isoelectric points (pI) ranged from 6 to 11. Recombinant His_6_-tagged proteins were purified using the TALON resin IMAC (immobilised metal ion affinity chromatography) system, according to the manufacturer’s instructions. Unbound or loosely bound non-target proteins were washed from the TALON resin column using TALON Buffer B (buffer A with 7.5 mM imidazole), and target proteins were eluted using TALON Buffer C (buffer A containing increasing concentrations of imidazole, namely 37.5 mM, 75 mM, and 150 mM). Eluted proteins were concentrated and exchanged into 50 mM sodium phosphate pH 6.5 using Amicon Ultra centrifugal filters with a molecular weight cut-off of 3 or 10 kDa (Merck Millipore, Sweden). SDS-PAGE analysis was used to verify the apparent molecular weight and purity of all recombinant proteins (see Supplementary Fig. 3). Photographs of SDS-PAGE gels were taken using a mobile phone camera and transferred into Adobe Illustrator 2022 for annotation.

### Macromolecular crystallography

Crystallisation conditions for *Cp*CBM92A and *Cp*CBM92B were screened using a Mosquito robot (SPT Labtech) and the JCSG+ screening kit (Molecular Dimensions, United Kingdom) in MRC sitting drop plates. Both proteins were dialysed into Tris (tris(hydroxymethyl)aminomethane, 50 mM) buffer at pH 8.0 containing NaCl (50 mM) prior to screening. Screens were prepared with a reservoir volume of 40 µL, and protein was mixed with reservoir solution in a 1:1 ratio in 0.6 µL drops. Within 2 weeks, crystals of varying quality were observed for both proteins in several of the conditions in the screen. Crystallisation conditions were optimised, and the final conditions used are listed in Supplementary Table 4. Crystals were mounted and flash frozen in liquid nitrogen in the absence of additional cryo-protectant. For ligand complexes of *Cp*CBM92B, crystals were soaked in reservoir solution containing a saturating amount of ligand for 1 min prior to flash freezing in liquid nitrogen. An initial dataset of *Cp*CBM92B diffracting to 2.1 Å was collected at the BioMAX beamline at MAX IV Laboratory (November 27, 2019) which was processed in XDS^62^ and the structure solved by molecular replacement using Balbes^63^ in CCP4 online^64^ which had identified and used PDB accession 3LLP, human fascin 1, as the search template. An initial model was built with ARP/wARP^65–69^. A subsequent data set of *Cp*CBM92B diffracting beyond 1.6 Å was collected at the BioMAX beamline at MAXIV Laboratory (March 27, 2020). Again this was processed with XDS^62^, and the solution was defined by rigid body refinement using Phenix Refine^70^ and the previously determined *Cp*CBM92B structure. Since the new dataset provided an improvement in resolution, only this dataset was pursued for further refinement and deposition. The datasets for *Cp*CBM92B-Glc and *Cp*CBM92B-G2 were processed by XDS^62^ and the structures determined by molecular replacement with Phaser^71^ in Phenix^72^ using the apo protein as the template. Datasets for *Cp*CBM92A and *Cp*CBM92B-S2 were anisotropic, and the data were elliptically truncated and corrected using the STARANISO server (http://staraniso.globalphasing.org)^73^. For all structures, Coot^74^ and Phenix Refine^70^ were used in iterative cycles of real space and reciprocal space refinement. The collection dates and locations, as well as the data collection, processing, and refinement statistics for all datasets can be found in Supplementary Table 5.

### Recombinant protein analysis

#### Differential scanning fluorimetry

To investigate protein stability at different temperature and pH conditions, *Cp*CBM92A and binding site variants thereof were analysed by differential scanning fluorimetry (DSF) using qPCR equipment (CFX96 Real-Time PCR detection system, Biorad, equipped with the software CFX Manager) with the SYPRO Orange dye (Thermo Fisher, Germany). Each reaction had a total volume of 25 µL, and a final protein concentration of 0.5–1.0 mg mL^−^^1^. Different buffers were used to analyse the protein melting point at different pH values: sodium citrate buffer (50 mM) was used at pH 4–5, while sodium phosphate buffer (50 mM) was used at pH 6–8. Lysozyme (Sigma-Aldrich) was used as a positive control. At least six replicates of each protein were analysed.

### Assessment of protein-carbohydrate interactions

#### Qualitative pull-down assays of binding to insoluble polysaccharides

Proteins were screened for the capacity to bind to insoluble or semi-soluble polysaccharides using a pull-down assay^75^. Briefly, 900 µL of polysaccharide at 5 g L^−^^1^ was mixed with 100 µL protein at ~0.5–3.0 g L^−^^1^ in sodium phosphate buffer (50 mM, pH 6.5) for 2 h at room temperature. The assays were incubated on a Stuart Rotator Disk turning at 30 rpm to provide continual gentle mixing throughout the assay. The assays were then centrifuged at 10,000 x *g* for 10 min and the supernatant was carefully collected into a fresh tube, without disturbing the pellet. Samples from the supernatants were analysed by SDS-PAGE. The absence of protein in the supernatant indicates binding to the insoluble polysaccharide, as the protein was ‘pulled down’ into the pellet during centrifugation. Ligands tested by pull-down assay were yeast β-glucan, pustulan, scleroglucan, birchwood xylan, lichenan, mixed linkage barley β-glucan, curdlan, oat spelt xylan, beechwood xylan, β-chitin, chitosan, ivory nut mannan, Avicel crystalline cellulose, starch, α-chitin, and shrimp chitin.

#### Depletion isotherms of binding to insoluble polysaccharides

Using 2 mL Eppendorf tubes, 20 µL of polysaccharide at 10 g L^−^^1^ was mixed with 0–80 µL protein at 500 ± 150 µM in 50 mM sodium phosphate buffer pH 6.5. Samples were incubated for 16 h at 4 °C with rotation at 20 rpm/min. Controls with protein but no ligand were performed to ensure that precipitation or other forms of protein loss did not occur during the assay. After incubation, samples were centrifuged at 4 °C, 10,000 x g for 10 min. The protein concentration in the supernatant was measured by nanodrop (A280) at least 3 times, without disturbing the pellets. The average of the three measured values was used for the determination of protein concentration, to determine the amounts of free and bound protein. Each data point represents an independent measurement. To ensure relevance and reproducibility, the data were generated using two preparations of proteins, and at least two independent technical replicates of the assay at each protein concentration were performed on different calendar dates.

#### Affinity gel electrophoresis to assay binding to soluble polysaccharides

To investigate the effect of different soluble polysaccharides on the migration rate of CBM92 domains under non-denaturing conditions, 1% (wt/vol) laminarin or carrageenan was incorporated into polyacrylamide gels prepared according to the Laemmli gel system^76^, but without SDS. The separating gel contained 10% acrylamide, and the stacking gel contained 4 % acrylamide (Invitrogen™ SureCast™). 20 μg of protein samples were loaded onto the gel in a standard loading buffer without SDS. Bovine serum albumin (BSA) served as the negative control since it does not bind to any polysaccharides. Gels were also prepared without polysaccharide to establish the baseline migration for each protein. Electrophoresis was conducted at 100 V for 4 h at 4 °C. The proteins were then visualised by staining with InstantBlue Protein Stain (Abcam).

#### Isothermal titration calorimetry (ITC) to assay binding to mono- and di-saccharides

Biomolecular interaction studies were performed using isothermal titration calorimetry (ITC)^77^, using a MicroCal iTC200 (Malvern Panalytical, Sweden). In each assay, ligand (5 mM) in sodium phosphate buffer (50 mM, pH 6.5) was used as a titrant against *Cp*CBM92A protein at ~100–200 µM. Each experimental run comprised 16 injections of 2 µL, with an injection flow rate of 0.5 μL s^−^^1^ and 100–120 s spacing between injections. The resulting titration thermograms were integrated using Origin 7 and curves were fitted to a single site model (N was set to 3) using chi-square testing. The ligands analysed in this way were d-glucose monosaccharide, β−1,4-linked cellobiose, β-1,3-linked laminaribiose, β-1,2-linked sophorose, and β−1,6-linked gentiobiose. The software Origin 2019 (OriginLab) was used to observe and export ITC data.

#### Bio-layer interferometry (BLI) to assay binding to oligosaccharides and soluble polysaccharides

Ni-NTA sensors were used for quantifying glycan-protein interactions. In a manner conceptually similar to IMAC protein purification, the tip of the Ni-NTA sensor binds to the His_6_ tag on recombinant proteins. BLI sensors coated with Ni-NTA (NTA) biosensors were purchased from Sartorius (Umeå, Sweden). They were immersed for 10 min in kinetic buffer (50 mM NaH_2_PO_4_, 50 mM NaCl, 10 mM imidazole, pH 6.5) before functionalisation. The sensors were then dipped in the kinetic buffer for another 5 min, then dipped in a solution of protein (1.2 μM) for 10 min. For every batch of analyses, one sensor was always dipped in no-protein buffer, as a reference control. To stabilise the captured CBMs, the sensors were then dipped in crosslinking reagent (0.1 M 1-ethyl-3-(3-dimethylaminopropyl)carbodiimide, 0.025 M N-hydroxysuccinimide in H_2_O) for 1 min, and then in quenching reagent (1 M ethanolamine, pH 8.5) for 1 min. After rinsing in kinetic buffer for 2 min, the sensors were ready for the sugar binding assay. The assay was initiated by dipping the sensors in different concentrations of oligosaccharide or polysaccharide for 10 min for each association step, and for 10 min in kinetic buffer for each dissociation step. The interferometry variation during ligand association/dissociation steps were analysed in real-time. The sensors were regenerated by dipping in glycine (10 mM), pH 1.7, and then in NiCl_2_ (10 mM) in water. Each sensor was reused up to 3 times. Reference sensors were used as blank for each batch of experiments to subtract the non-specific adsorption from the raw data. The sensorgrams were fitted using a single binding model (1:1) for S5, and the data were analysed using the Octet Software Version 10.0 on the kinetic parameters of binding interactions. Many different concentrations of oligosaccharides and polysaccharides were tested, and only those experiments showing apparent binding with wild type *Cp*CBM92A were pursued. Data were visualised using MatLab version E2021a.

### Reporting summary

Further information on research design is available in the Nature Portfolio Reporting Summary linked to this article.

### Supplementary information

Supplementary Information

Reporting Summary

### Source data

Source data

## Data Availability

Accession codes for all proteins used in bioinformatic or biochemical analysis are provided in Supplementary Tables 1 and 2. Coordinates and structure factors for *Cp*CBM92A, *Cp*CBM92B, and *Cp*CBM92B in complex with glucose, gentiobiose, and sophorose have been deposited in the RCSB Protein Data Bank under accession codes 7ZOI, 7ZOH, 7ZON, 7ZOO, and 7ZOP, respectively. All other data generated or analysed during this study are included in this published article, the Supplementary Information, and the source data files. Source data are provided with this paper.
