# Supplementary material for: Structural and biochemical analysis of family 92 carbohydrate-binding modules uncovers multivalent binding to β-glucans
Source: Nat Commun. 2024 Apr 23;15:3429. doi: 10.1038/s41467-024-47584-y (PMC11039641; doi:10.1038/s41467-024-47584-y)
Supplement: Supplementary file 1 — Supplementary Information [file 41467_2024_47584_MOESM1_ESM.pdf]

Supplementary figures

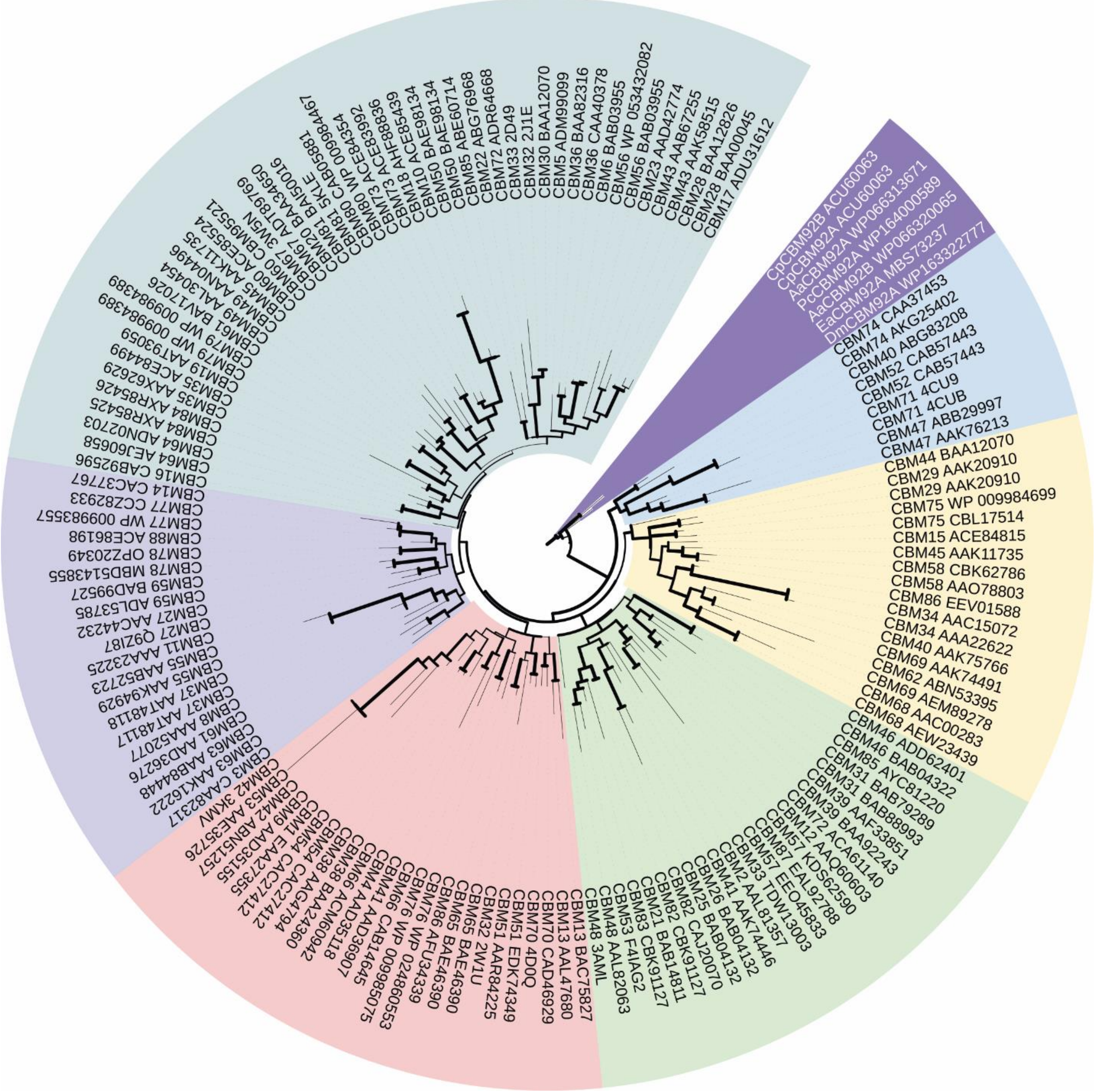

Supplementary Figure 1. Family CBM92 is shown to be clearly distinct from all other CBM families currently represented on the CAZy database. Seven CBM92 domains are included, compared with 1-3 proteins with crystal structure and/or known binding specificity from every other CBM family on the CAZy database at time of writing. Only the sequences of the CBM domains themselves were used to produce the alignment for this analysis. Proteins are labelled with CBM family annotation and NCBI or PDB accession numbers. See complete abbreviations and sequence accession number in **Supplementary Table 1**. The tree was produced by maximum likelihood using the iQtree online server. Bootstrap value is indicated by branch thickness.

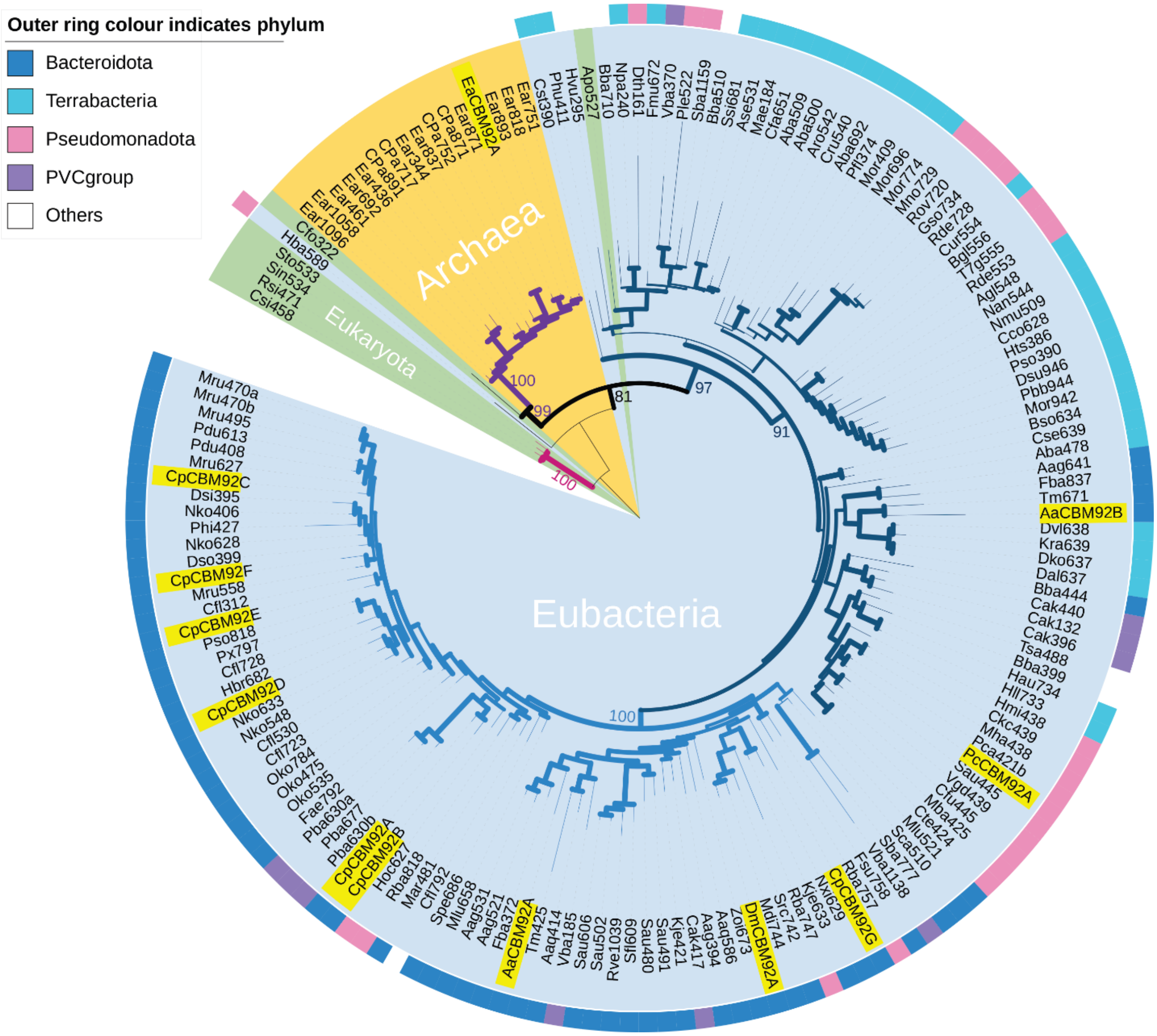

Supplementary Figure 2. Maximum likelihood phylogenetic analysis of the sequences of 164 CBM92 domains generated using the iQtree web server. Bootstrap values are indicated by branch thickness, and by numbers for the major distinct clades, whose branches are coloured to match their bootstrap value. The tree is displayed as a circle and is unrooted. Protein names are shown as an abbreviation of species names and their protein length. See complete abbreviations and sequence accession numbers in **Supplementary Table 1**. The sequences highlighted in yellow boxes were selected for further analysis.

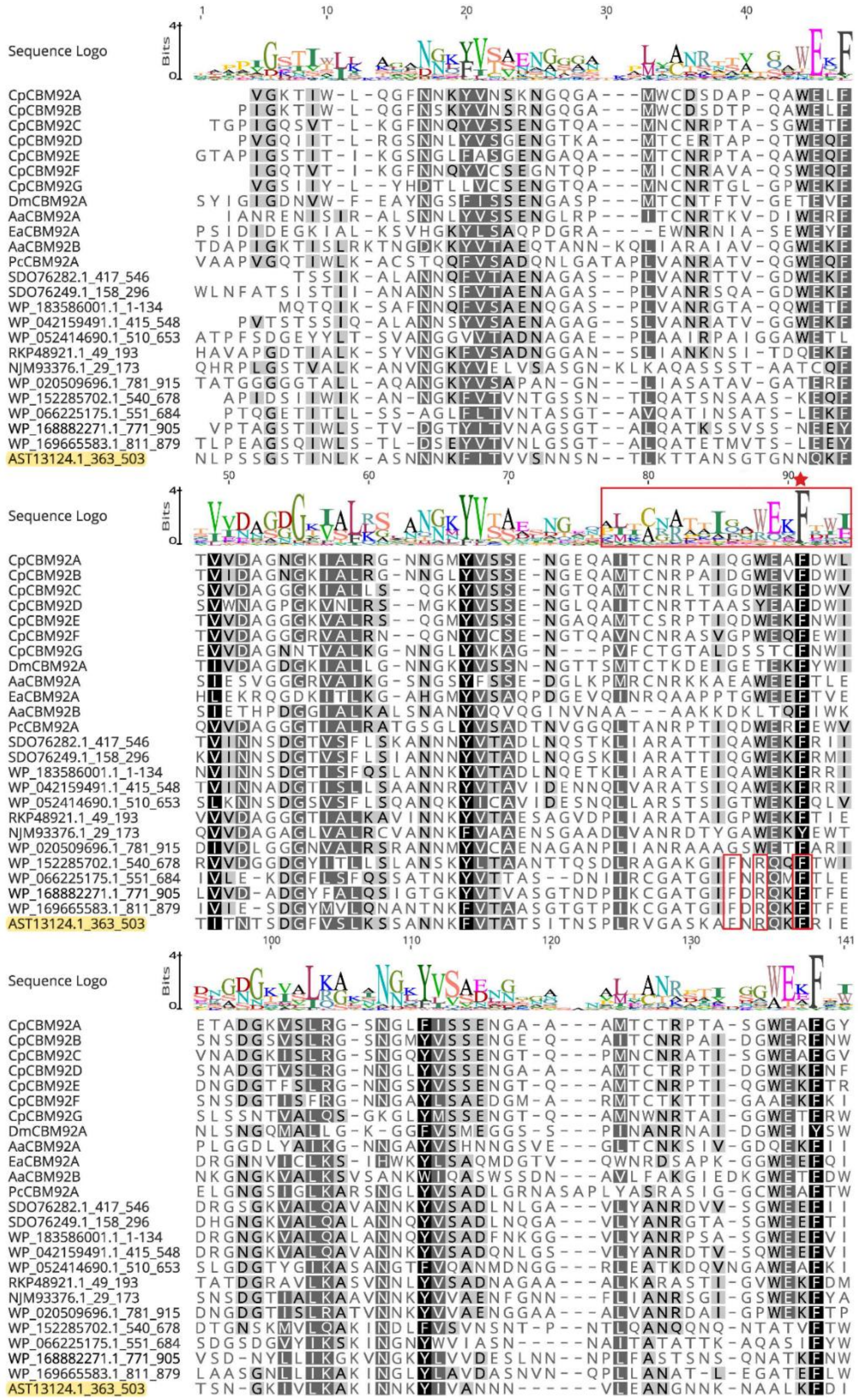

Supplementary Figure 3a. In the alignment, a carrageenan-binding CBM92 protein characterised by Mei *et al* (6) is highlighted in yellow. Two Phe (F) residues were proposed to be important for carrageenan binding in that protein, and these are highlighted in a red box in the sequences of known and putative carrageenan binders, as described by Mei et al. 2022. These sequences appear to represent a small sub-group within the family, as other members instead possess three WExF motifs, corresponding to three distinct binding sites, all of which can be functional in β-glucan binding provided the Trp is present.

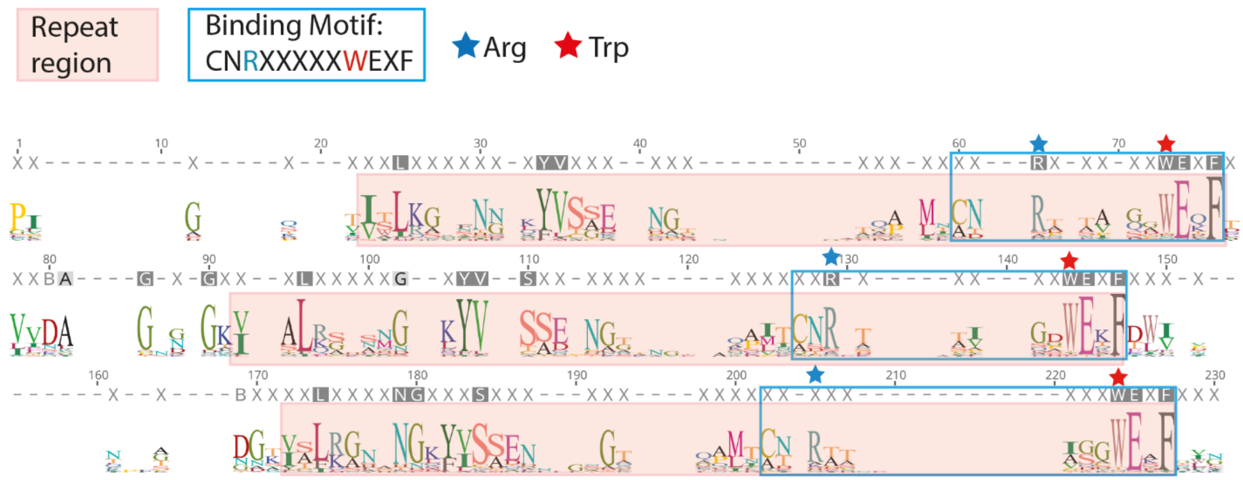

Supplementary Figure 3b. A consensus sequence logo derived from an alignment of 818 CBM92 domain sequences is shown beneath the alignment. The Trp and Arg involved in pustulan binding in the proteins we studied are indicated respectively by a red and a blue star. The logo shows that proteins with the three WExF domains dominate the consensus sequence for the family, as the alternative carrageen-binders are a small sub-set of the family.

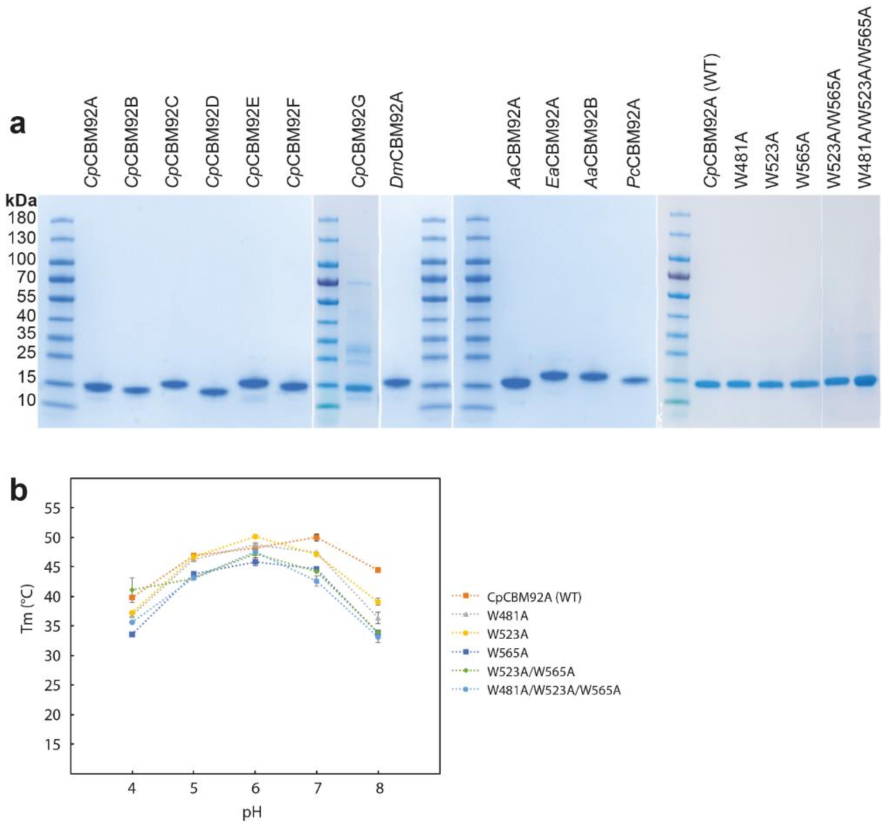

Supplementary Figure 4. (a) SDS-PAGE analysis shows successful production and purification of the CBM92 proteins analysed in this study. (b) DSF analysis shows that the variant forms of *Cp*CBM92A are as stable as the wild-type, displaying the same melting temperature (Tm) profiles at different pH values.

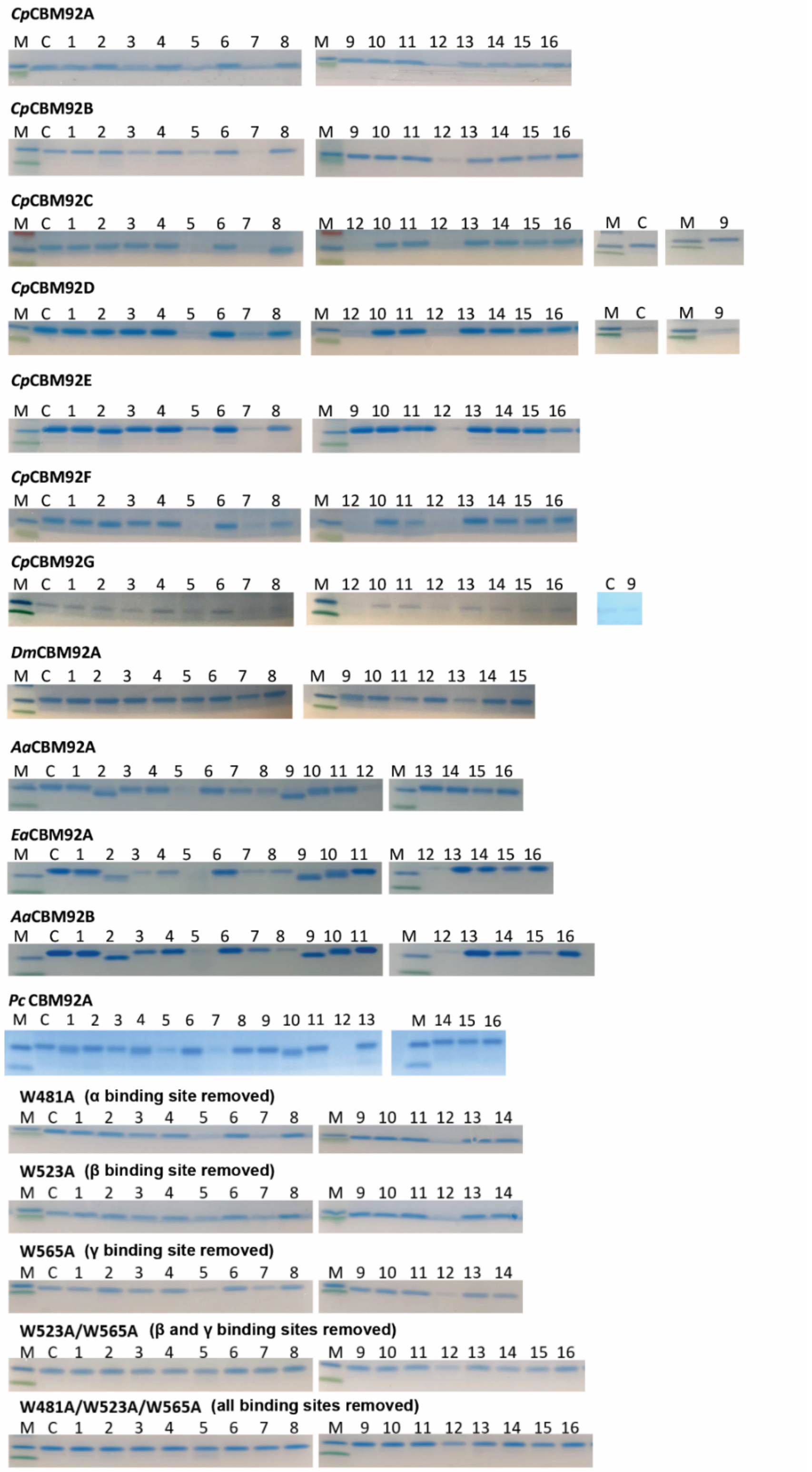

Supplementary Figure 5a. SDS-PAGE was performed on the supernatants collected from pull-down assays and indicates whether a protein-polysaccharide binding interaction has occurred. A band visible on the gel indicates that the protein remained in solution after mixing with the polysaccharide, which means no binding occurred. If a band is

absent from the gel, this indicates that the protein was “pulled down” from the solution during centrifugation because it was bound to the insoluble polysaccharide. The polysaccharides used to produce samples in the lanes indicated are as follows. C: control (no polysaccharide included). 1: Avicel cellulose. 2: curdlan. 3: lichenan. 4: barley β-glucan. 5: scleroglucan. 6: starch. 7: pustulan. 8: birchwood xylan. 9: β chitin. 10: α chitin. 11: shrimp shell chitin. 12: yeast β-glucan. 13: chitosan. 14: ivory nut mannan. 15: oat spelt xylan. 16: beechwood xylan. The first lane (M) in each gel shows molecular weight markers in green (bottom, 10 kDa) and blue (top, 15 kDa). The assay for each protein was performed three times, and the same results were obtained each time. One representative SDS-PAGE image is provided for each protein.

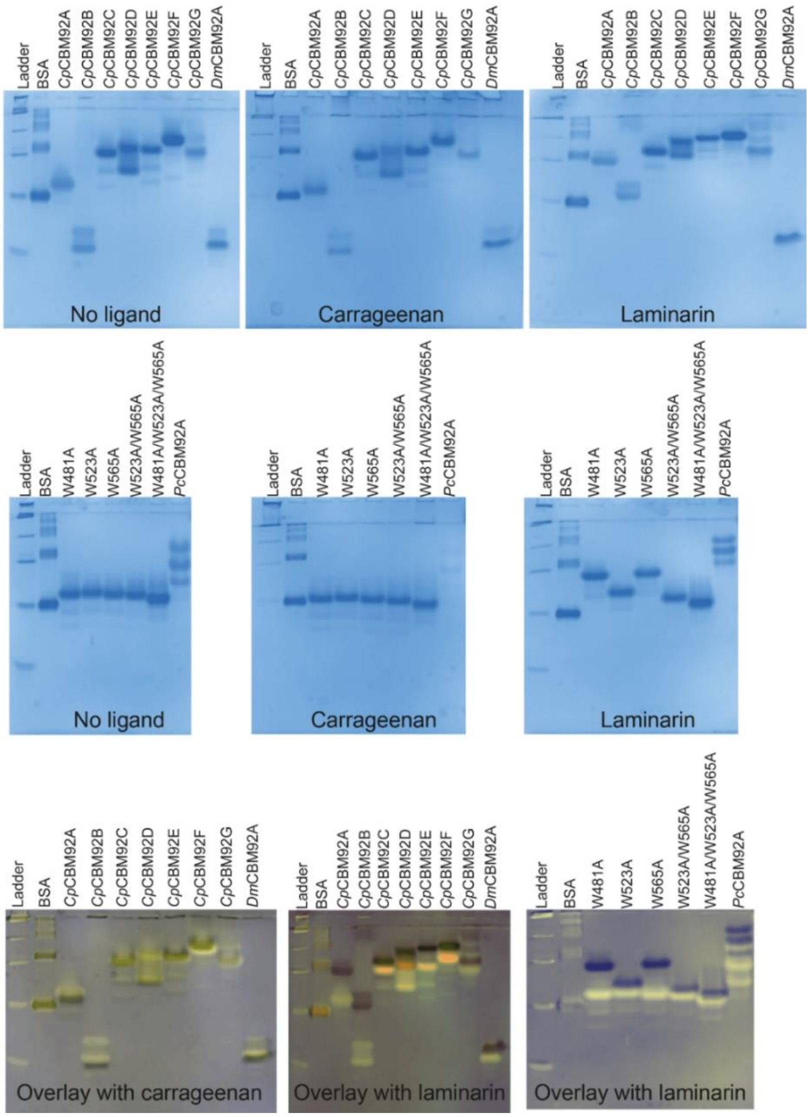

Supplementary Figure 5b. Affinity gel electrophoresis was performed to assay for binding to carrageenan and lamarin. Ligand was incorporated into NativePAGE gels at a concentration of 1 mg mL^{-1}. A total of 20 µg of protein was loaded in each well. A shift or retardation in migration for a protein when ligand is present, compared to when ligand is absent, indicates qualitatively that binding is occurring. Proteins not shown here could not be analysed using this method due to a very high isoelectric point. Top: diverse wild type proteins are screened for binding. Middle: *Pc*CBM92A and variant forms of *Cp*CBM92A are screened for binding. W481A = α binding site variant; W523A =

β site variant; W565A = γ site variant; W523A/W565A = β/γ sites variant; W481A/W523A/W565A = triple binding site variant. Note that the bands for *Pc*CBM92A are very faint on the carrageenan gel, but they are located in the same positions as in the ligand-free gel. Bottom: overlaid ligand-free and ligand-containing gels confirm binding to laminarin but not to carrageenan.

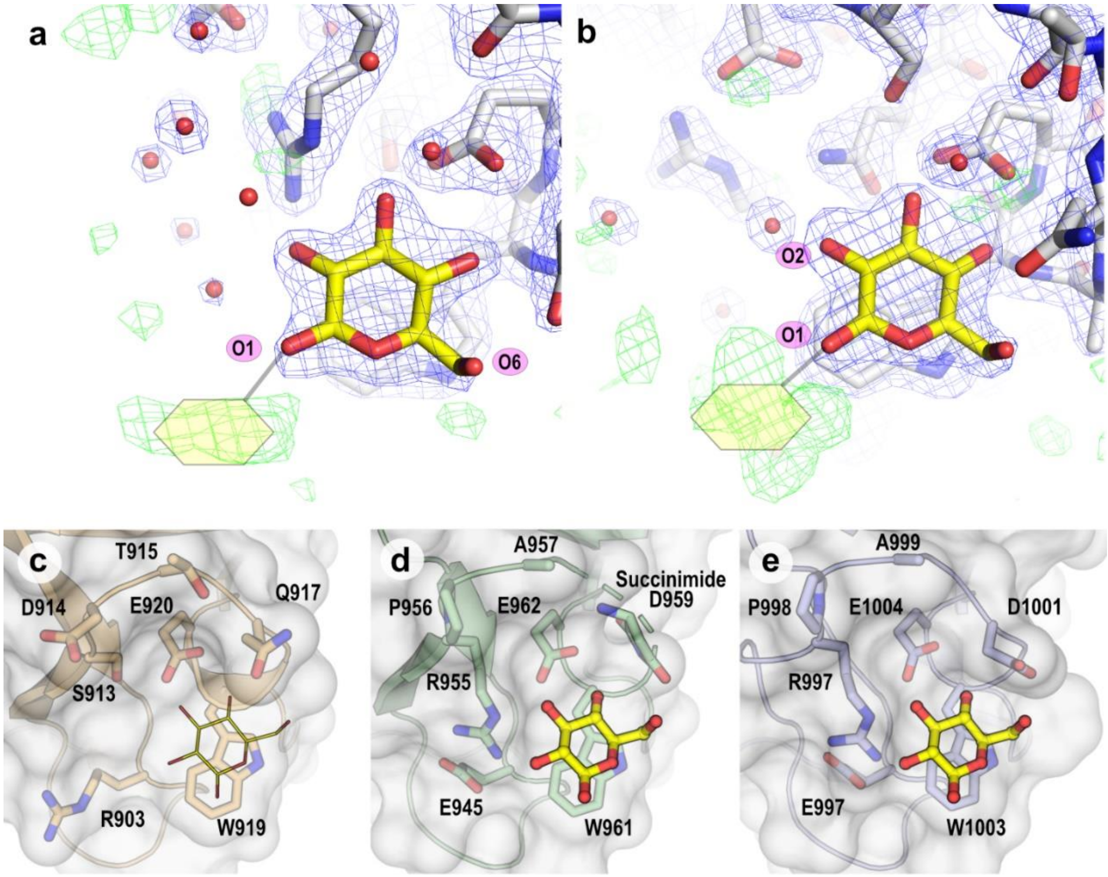

Supplementary Figure 6. *Cp*CBM92A in complex with gentiobiose and sophorose. Structures of *Cp*CBM92A in complex with (a) gentiobiose in the β-subdomain and (b) sophorose in the α-subdomain with both showing carbon atoms in grey, oxygen in red, and nitrogen in blue. In each, only the non-reducing glucose unit of the disaccharide, shown in yellow carbons, was able to be modelled into the binding cleft. Density from the 2Fo-Fc electron density maps contoured at 1.5σ level are shown as a blue mesh and positive density from Fo-Fc maps contoured at 2.5σ level is shown as green mesh. In both, considerable positive density in the Fo-Fc maps were present beyond the O1 of the non-reducing glucose unit but insufficient 2Fo-Fc electron density was present to reliably model the reducing end glucose of the disaccharide. The expected position of the unresolved glucose units of the disaccharides are shown as transparent yellow hexagons, and the positions where extensions are possible for longer gentio (β-1,6) and sophoro (β-1,2) oligosaccharides are highlighted in magenta. Panels (c-e) show a comparison of the binding sites in *Cp*CBM92B. The α (c), β (d), and γ (e) subsites are shown in complex with glucose, shown as yellow sticks. The glucose in the α subsite (shown as lines) is placed based on structural alignment to the glucose units experimentally observed in the β and γ subsites. Notably, a serine (S913) is found in the α subsite where an arginine is observed in both the β and γ subsites (respectively R955 and R997).

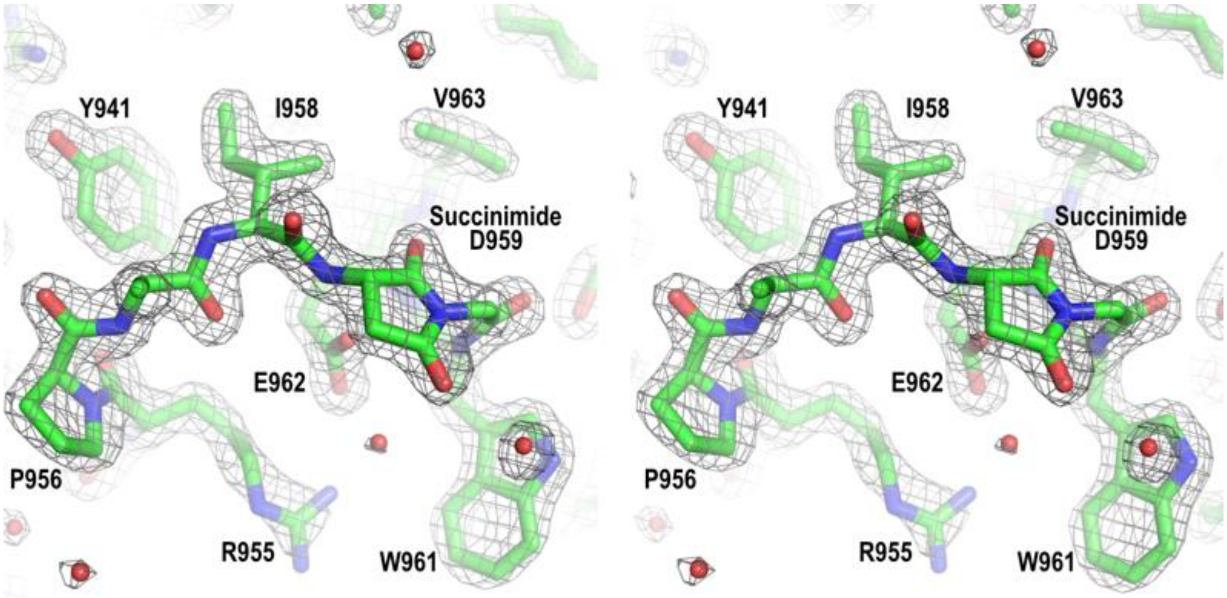

Supplementary Figure 7. Stereo image of a portion of the electron density map of *Cp*CBM92A. The model of the protein around the binding cleft in the β-subdomain, highlighting the presence of the succinimide, is shown with carbon atoms in green, oxygen in red, and nitrogen in blue. Water molecules are shown as red spheres. Density from the 2Fo-Fc electron density map contoured at 1.5σ level is shown as a grey mesh.

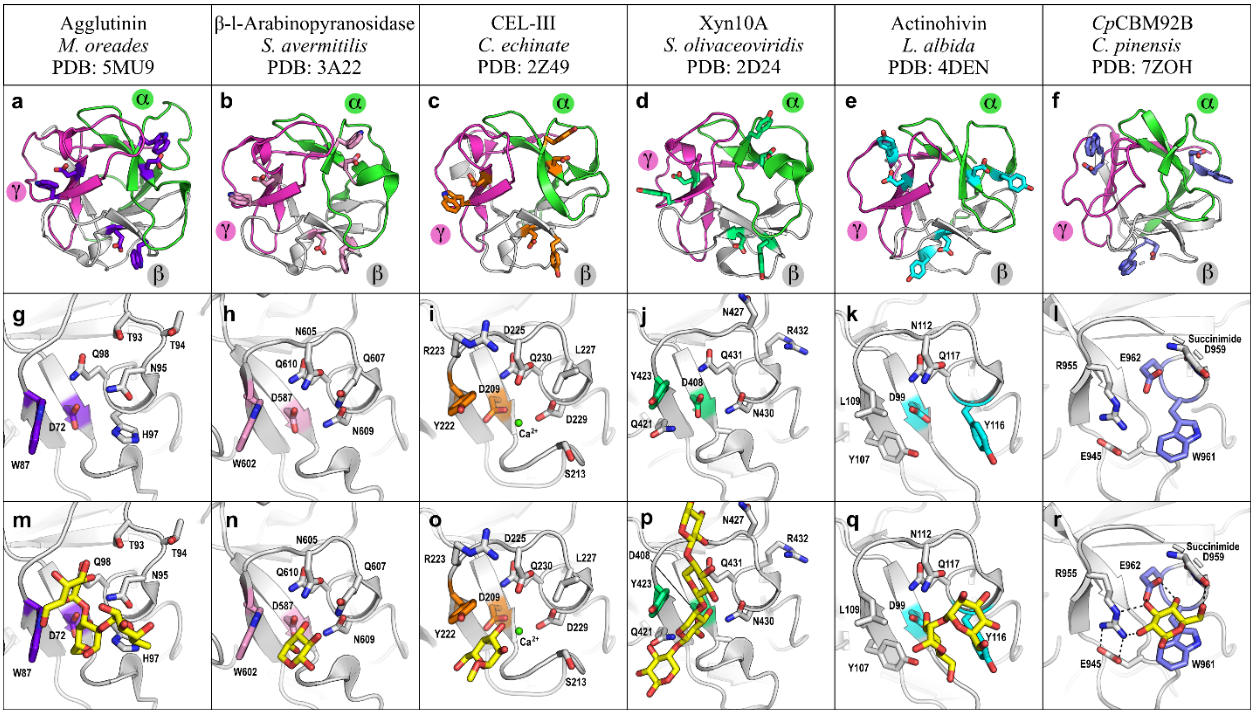

Supplementary Figure 8. Comparison of *Cp*CBM92B with CBM13 members. The overall structures of the β-trefoil domains from (a,g,m) agglutinin from *Marasmius oreades* (PDB: 5MU9), (b,h,n) β-L-arabinopyranosidase (GH27) from *Streptomyces avermitilis* (PDB: 3A22), (c,i,o) CEL-III from *Cucumaria echinate* (PDB: 2Z49), (d,j,p) xylanase

Xyn10A (GH10) from *Streptomyces olivaceoviridis* E-86 (PDB: 2D24), (e,k,q) actinohivin from *Longispora albida* K97-0003T (PDB: 4DEN), and (f,l,r) CpCBM92B. The subdomains are coloured and the aromatic and acidic residues supporting ligand binding in each are shown differentially coloured. Panels g-l show the binding sites in the β-subdomain of each protein and panels m-r show the same site with their bound ligands (yellow sticks). The ligands shown in each panel are: (m) a trisaccharide of fucose-α-1,2-galactose-α-1,3-galactose, (n) arabinopyranose (o) methyl α-galactoside, (p) xylotetraose, (q) α-1,2-mannobiose, and (r) glucose.

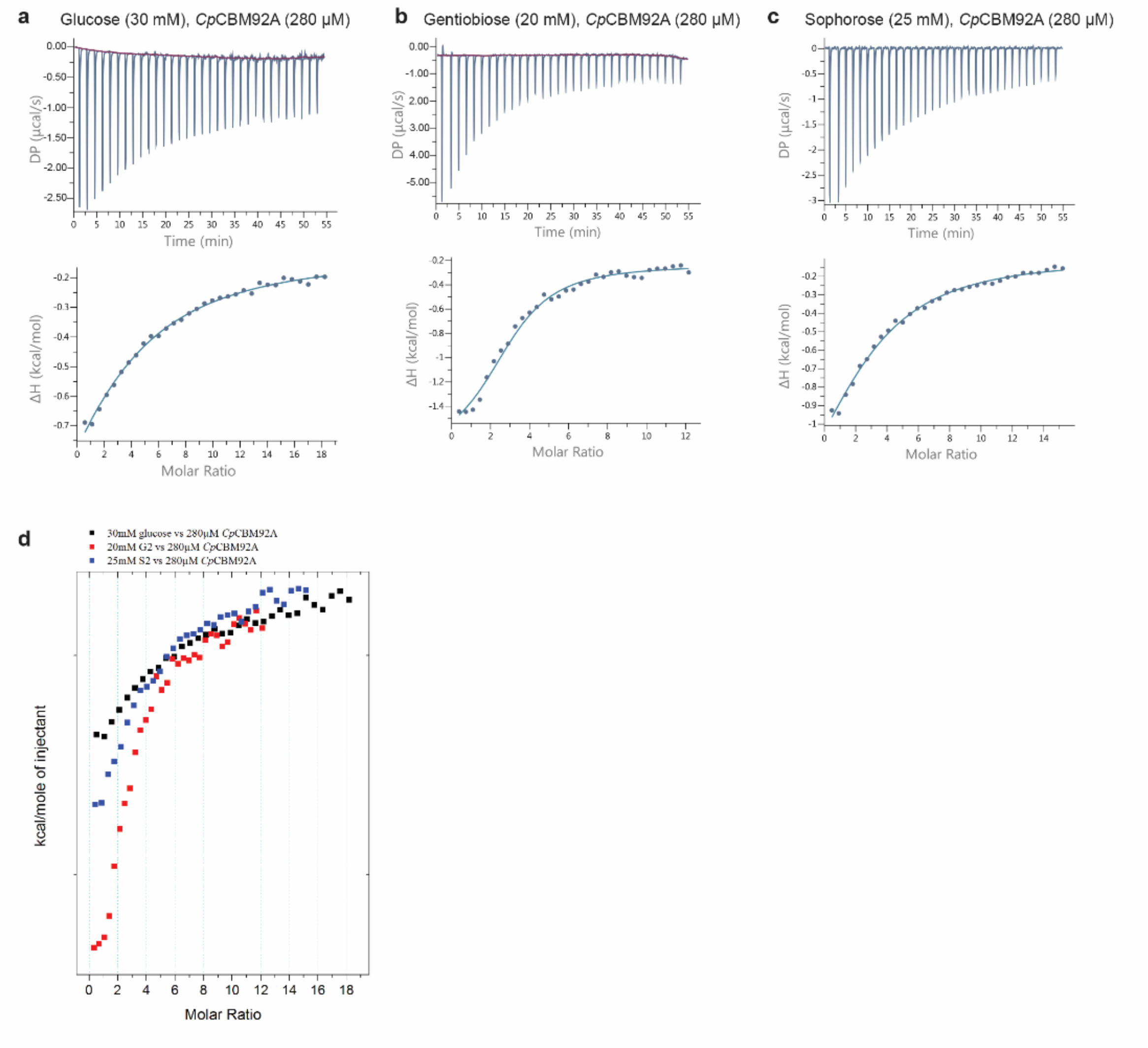

Supplementary Figure 9. Ligand binding interactions of *Cp*CBM92A, demonstrated by isothermal titration calorimetry (ITC). The ITC data were fitted to a single site binding model for all ligands. As the wild type *Cp*CBM92A protein has three binding sites, N was set to 3. (a) Glucose (30 mM) binding to *Cp*CBM92A (280 µM). (b) Gentiobiose (20 mM) binding to *Cp*CBM92A (280 µM). (c) Sophorose (25 mM) binding to *Cp*CBM92A (280 µM). In panels (a-c), data from a control experiment are shown in blue, where ligand was injected into buffer. Panels (d-f) show curve-fitting analyses of the same data. (d) An overlay provides a visual comparison of binding to the three ligands.

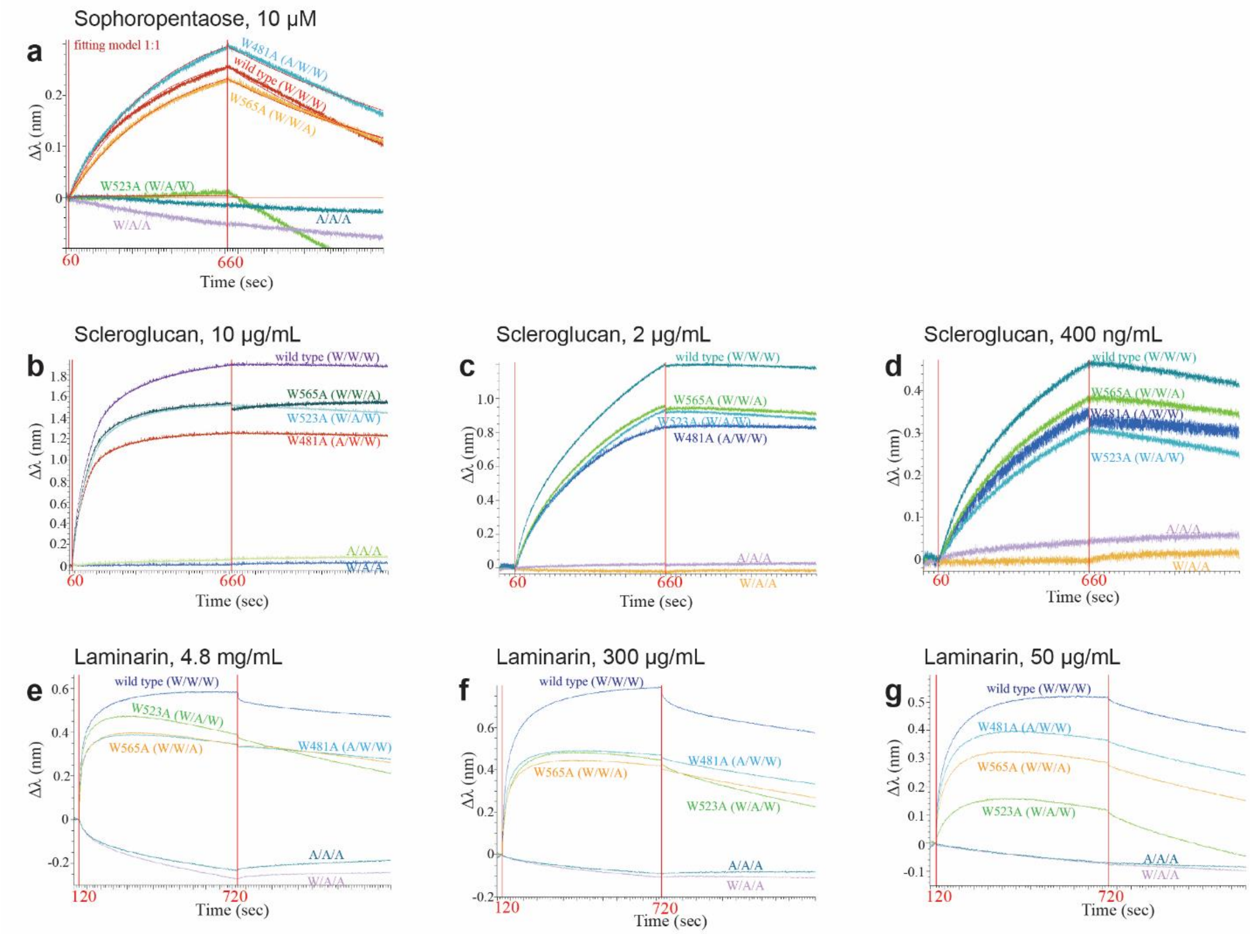

Supplementary Figure 10. Binding of *Cp*CBM92A and its variants to different polysaccharides and oligosaccharides as determined by BLI. All proteins were pre-immobilised on the sensor at 20 µg/mL. The graphs depict wild type and variant forms of *Cp*CBM92A binding to ligand at the following concentrations: (a) sophoropentaose at 10 µM. (b) scleroglucan at 10 µg/mL (c) scleroglucan at 2 µg/mL (d) scleroglucan at 400 ng/mL (e) laminarin at 4.8 mg/mL (f) laminarin at 300 µg/mL (g) laminarin at 50 µg/mL.

Supplementary tables

Supplementary Table 1. Gene accession numbers, species name, and tree node label used on phylogenetic analysis shown in **Figure 2**. Tree node labels comprise an abbreviation of the species name and the number of amino acids in the full-length protein. Domains that were characterised within this study are indicated by an asterisk (*) after the tree node label.

Tree node label	Species	NCBI accession No.
**AaCBM92A ***	*Aquimarina aggregata*	WP_066313671.1
**AaCBM92B ***	*Aquimarina aggregata*	WP_066320065.1
**Aag394**	*Aquimarina aggregata*	WP_066313446.1
**Aag521**	*Aquimarina aggregata*	WP_066310828.1
**Aag531**	*Aquimarina aggregata*	WP_157766178.1
**Aag641**	*Aquimarina aggregata*	WP_066315015.1
**Aaq414**	*Algibacter aquaticus*	WP_069828882.1
**Aaq586**	*Algibacter aquaticus*	WP_069829724.1
**Aba478**	*Armatimonadetes bacterium*	NMC82449.1
**Aba500**	*Actinobacteria bacterium*	TMM36148.1
**Aba509**	*Acidimicrobiia bacterium*	MBA3268160.1
**Aba692**	*Actinobacteria bacterium*	TMM30772.1
**Agl548**	*Actinoplanes globisporus*	WP_020509689.1
**Apo527**	*Apiotrichum porosum*	XP_028473893.1
**Aro542**	*Actinospica robiniae*	WP_063627936.1
**Ase531**	*Anaeromicrobium sediminis*	WP_095133838.1
**Bba399**	*Blastocatellia bacterium*	MBO0723753.1
**Bba444**	*Blastocatellia bacterium*	MBL8206846.1
**Bba510**	*Blastocatellia bacterium*	PWT82357.1
**Bba710**	*Blastocatellia bacterium*	MBL8206846.1
**Bgl556**	*Burkholderia gladioli*	WP_186190248.1
**Bso634**	*Bacillus solimangrovi*	OEH91538.1
**Cak132**	*Coraliomargarita akajimensis* DSM 45221	ADE54029.1
**Cak396**	*Coraliomargarita akajimensis* DSM 45221	ADE54011.1
**Cak417**	*Coraliomargarita akajimensis* DSM 45221	ADE53100.1
**Cak440**	*Coraliomargarita akajimensis* DSM 45221	ADE53121.1
**Cco628**	*Capsulimonas corticalis*	WP_119324998.1
**Cfa651**	*Coleofasciculus sp.* FACHB-129	WP_190676778.1
**Cfl312**	*Chryseolinea flava*	WP_112749069.1
**Cfl474**	*Chryseolinea flava*	WP_112748658.1
**Cfl530**	*Chryseolinea flava*	WP_112748659.1
**Cfl723**	*Chryseolinea flava*	RAW00633.1
**Cfl728**	*Chryseolinea flava*	WP_185147044.1
**Cfo322**	*Coptotermes formosanus*	AEW67353.1

Tree node label	Species	NCBI accession No.
**Cfu445**	*Cystobacter fuscus*	WP_095989217.1
**Ckc439**	*Comamonas sp.* KCTC 72670	WP_191267545.1
**CPa717**	*Candidatus Poseidoniales archaeon*	RCH73252.1
**CPa752**	*Candidatus Poseidoniales archaeon*	DAC55731.1
**CPa871**	*Candidatus Poseidoniales archaeon*	DAC30974.1
**CPa891**	*Candidatus Poseidoniales archaeon*	DAC10775.1
**CpCBM92A (and B) ***	*Chitinophaga pinensis* DSM 2588	ACU60063.1
**CpCBM92C ***	*Chitinophaga pinensis* DSM 2588	ACU61803.1
**CpCBM92D ***	*Chitinophaga pinensis* DSM 2588	ACU61215.1
**CpCBM92E ***	*Chitinophaga pinensis* DSM 2588	ACU62541.1
**CpCBM92F ***	*Chitinophaga pinensis* DSM 2588	ACU61621.1
**CpCBM92G ***	*Chitinophaga pinensis* DSM 2588	ACU61949.1
**Cru540**	*Catenulispora rubra*	WP_194909551.1
**Cse639**	*Chengkuizengella sediminis*	WP_162037177.1
**Csi458**	*Camellia sinensis*	XP_028082931.1
**Cst390**	*Cylindrospermum stagnale*	WP_172642182.1
**Cte424**	*Corallococcus terminator*	WP_120540014.1
**Cur554**	*Cellulomonas sp.* URHD0024	WP_028051117.1
**Dal637**	*Dictyobacter alpinus*	WP_126631529.1
**Dko637**	*Dictyobacter kobayashii*	WP_126557407.1
**DmCBM92A ***	*Draconibacterium sp.* GM2-18	WP_163322777.1
**Dsi395**	*Dinghuibacter silviterrae*	WP_133997709.1
**Dso399**	*Deminuibacter soli*	WP_116848117.1
**Dsu946**	*Dactylosporangium sucinum*	WP_190249133.1
**Dth161**	*Dichotomicrobium thermohalophilum*	WP_119060661.1
**Dvl638**	*Dictyobacter vulcani*	WP_151757874.1
**EaCBM92A ***	*Euryarchaeota archaeon*	MBS73237.1
**Ear1058**	*Euryarchaeota archaeon*	MBV23397.1
**Ear1096**	*Euryarchaeota archaeon*	MBC64308.1
**Ear344**	*Euryarchaeota archaeon*	MBJ29332.1
**Ear436**	*Euryarchaeota archaeon*	MBH33816.1
**Ear461**	*Euryarchaeota archaeon*	MBJ36561.1
**Ear692**	*Euryarchaeota archaeon*	MAN07855.1
**Ear751**	*Euryarchaeota archaeon*	MAY05287.1
**Ear818**	*Euryarchaeota archaeon*	MBP65661.1
**Ear837**	*Euryarchaeota archaeon*	MBM54141.1
**Ear871**	*Euryarchaeota archaeon*	MBA86910.1
**Ear893**	*Euryarchaeota archaeon*	RAH12883.1
**Fae792**	*Fibrella aestuarina*	WP_083891454.1

Tree node label	Species	NCBI accession No.
**Fba372**	*Flavobacteriaceae bacterium* M625	TPN87735.1
**Fba837**	*Flavobacteriaceae bacterium* M625	TPN83373.1
**Fmu672**	*Fischerella muscicola*	WP_016861236.1
**Fsu758**	*Flavobacterium subsaxonicum*	WP_051200144.1
**Gso734**	*Geomonas soli*	WP_124539163.1
**Hau734**	*Herpetosiphon aurantiacus* DSM 785	ABX04162.1
**Hba589**	*Hyphomicrobiaceae bacterium*	TXH09896.1
**Hbr682**	*Hymenobacter sp.* BRD128	WP_173117794.1
**Hll733**	*Herpetosiphon llansteffanensis*	WP_110518824.1
**Hmi438**	*Hyalangium minutum*	WP_044181817.1
**Hoc627**	*Haliangium ochraceum*	WP_012831313.1
**Hts386**	*Hamadaea tsunoensis*	WP_027344156.1
**Hvu295**	*Hydra vulgaris*	AAZ31366.1
**Kje421**	*Kordia jejudonensis*	WP_053002293.1
**Kje633**	*Kordia jejudonensis*	WP_046756685.1
**Kra639**	*Ktedonobacter racemifer*	WP_007909656.1
**Mae184**	*Microcystis aeruginosa*	WP_139374347.1
**Mar481**	*Marinoscillum sp.* 108	WP_159579538.1
**Mba425**	*Myxococcaceae bacterium*	RYZ39347.1
**Mdi744**	*Mangrovibacterium diazotrophicum*	WP_120272090.1
**Mha438**	*Myxococcus hansupus*	WP_002636775.1
**Mlu521**	*Marivirga lumbricoides*	PTB97274.1
**Mlu658**	*Marivirga lumbricoides*	WP_188463823.1
**Mno729**	*Mitsuaria noduli*	OWQ45395.1
**Mor409**	*Micromonospora orduensis*	WP_139587827.1
**Mor696**	*Micromonospora orduensis*	WP_139588083.1
**Mor774**	*Micromonospora orduensis*	WP_139586123.1
**Mor942**	*Micromonospora orduensis*	WP_139584258.1
**Mru470a**	*Mucilaginibacter rubeus*	WP_129569879.1
**Mru470b**	*Mucilaginibacter rubeus*	WP_112568850.1
**Mru495**	*Mucilaginibacter rubeus*	QEM12674.1
**Mru558**	*Mucilaginibacter rubeus*	WP_112575541.1
**Mru627**	*Mucilaginibacter rubeus*	QEM13197.1
**Nan544**	*Nonomuraea angiospora*	MBE1592301.1
**Nko406**	*Niastella koreensis*	WP_014216967.1
**Nko548**	*Niastella koreensis*	WP_014216945.1
**Nko628**	*Niastella koreensis*	WP_014216968.1
**Nko633**	*Niastella koreensis*	WP_014221059.1

Tree node label	Species	NCBI accession No.
**Nmu509**	*Nakamurella multipartita*	WP_138180402.1
**Npa240**	*Nostoc parmelioides*	WP_190572743.1
**Nxi629**	*Nonlabens xiamenensis*	WP_124979265.1
**Oko475**	*Ohtaekwangia koreensis*	WP_079685365.1
**Oko535**	*Ohtaekwangia koreensis*	WP_079685366.1
**Oko784**	*Ohtaekwangia koreensis*	WP_079690368.1
**Pba630a**	*Phycisphaerales bacterium*	HCD29691.1
**Pba630b**	*Phycisphaerales bacterium*	MAV55595.1
**Pba677**	*Phycisphaerae bacterium*	MAW42132.1
**Pbb944**	*Plantactinospora sp.* BB1	WP_107259782.1
**Pca421b**	*Pyxidicoccus sp.* CA060A	WP_164018937.1
**PcCBM92A ***	*Pyxidicoccus sp.* CA060A	WP_164000589.1
**Pdu408**	*Pedobacter duraquae*	WP_133555761.1
**Pdu613**	*Pedobacter duraquae*	WP_133557106.1
**Pfl374**	*Phytohabitans flavus*	WP_173035271.1
**Phi427**	*Pseudarcicella hirudinis*	SFP22661.1
**Phu411**	*Paenibacillus hunanensis*	WP_188774785.1
**Ple522**	*Paucimonas lemoignei*	WP_132259237.1
**Pso390**	*Plantactinospora soyae*	MBE1491319.1
**Pso818**	*Paraflavitalea soli*	AXY74800.1
**Px797**	*Pseudoflavitalea sp.* X16	WP_167290596.1
**Rba747**	*Rickettsiales bacterium*	MBR07351.1
**Rba757**	*Rickettsiales bacterium*	MBR07353.1
**Rba818**	*Rickettsiales bacterium*	MBR07521.1
**Rde553**	*Roseateles depolymerans*	WP_058935987.1
**Rde728**	*Roseateles depolymerans*	ALV08126.1
**Rov720**	*Rhizobacter sp.* OV335	WP_073469098.1
**Rsi471**	*Rhododendron simsii*	KAF7129156.1
**Rve1039**	*Reichenbachiella versicolor*	WP_109831106.1
**Sau445**	*Stigmatella aurantiaca*	WP_002612833.1
**Sau480**	*Saccharicrinis aurantiacus*	WP_068475139.1
**Sau491**	*Saccharicrinis aurantiacus*	WP_075602374.1
**Sau502**	*Saccharicrinis aurantiacus*	WP_075603264.1
**Sau606**	*Saccharicrinis aurantiacus*	WP_075603263.1
**Sba1159**	*Syntrophaceae bacterium*	NWF54575.1
**Sba777**	*Saprospirales bacterium*	HAQ39283.1
**Sca510**	*Saccharicrinis carchari*	WP_142533534.1
**Sfl609**	*Sediminitomix flava*	WP_109621230.1
**Sin534**	*Sesamum indicum*	XP_011097508.1

Tree node label	Species	NCBI accession No.
**Spe686**	*Sinomicrobium pectinilyticum*	WP_123218104.1
**Src742**	*Sunxiuqinia sp.* RC1_OXG_1F	WP_159523523.1
**Ssi681**	*Symploca sp.* SIO2E6	NET60575.1
**Sto533**	*Senna tora*	KAF7834451.1
**T7g555**	*Trinickia sp.* 7GSK02	WP_136892366.1
**Tm425**	*Tenacibaculum sp.* M341	WP_132722194.1
**Tm671**	*Tenacibaculum sp.* M341	WP_132722652.1
**Tsa488**	*Terriglobus saanensis*	WP_013567755.1
**Vba1138**	*Verrucomicrobia bacterium*	NCX47021.1
**Vba185**	*Verrucomicrobia bacterium*	MBD99715.1
**Vba370**	*Verrucomicrobia bacterium*	PYK05847.1
**Vgd439**	*Vitiosangium sp.* GDMCC 1.1324	WP_108075538.1
**Zoi673**	*Zobellia sp.* OII3	WP_088696129.1

Supplementary Table 2. Constructs generated in this study, and the cloning strategies used to produce them. CBM92 domains were cloned from large multi-modular genes. The range of nucleotides specified therefore refers to the fragment that was cloned from the full-length gene. The size (kDa) refers to the resulting recombinant CBM92 protein. gDNA = genomic DNA.

**Organism**	**Genbank accession no. and range of amino acids included in construct**	**Protein name in this paper**	**Size (kDa)**	**Cloning strategy***	**Primer pairs**
Chitinophaga pinensis	ACU60063.1 448-576	*Cp*CBM92A	16.2	Commercial synthesis	none
Chitinophaga pinensis	ACU60063.1 888-1012	*Cp*CBM92B Used in structural analysis	15.4	Commercial synthesis	none
Chitinophaga pinensis	ACU60063.1 888-1012	*Cp*CBM92B Used in NativePAGE	15.4	Cloned from gDNA into pLATE31	5’-AGAAGGAGATATAACTATGCCGATCGGTAAAACGATCTGGTTACAG-3’ 5'-GTGGTGGTGATGGTGATGGCCAGTCAATGCTGTTGCTGCTGC-3’
Chitinophaga pinensis	ACU61803.1 476-603	*Cp*CBM92C	16.0	Cloned from gDNA into pET21a	5’-CTCGAGCTGATTGACGCCAA-3’ 5’-GGATCCACAGGACCTATCGGACAA-3’
Chitinophaga pinensis	ACU61215.1 414-542	*Cp*CBM92D	14.9	Cloned from gDNA into pLATE31	5’-AGAAGGAGATATAACTATGCCGGTTGGCCAGATCATCACACTCAG-3’ 5’-GTGGTGGTGATGGTGATGGCCTGCTGGTCCGACGATCGTGAAATTG-3’
Chitinophaga pinensis	ACU62541.1 275-409	*Cp*CBM92E	16.5	Cloned from gDNA into pET21a	5’-GGATCCATCACCGATACACCTGGT-3’ 5’-CTCGAGGCGGGTGAATTTCTC-3’

**Organism**	**Genbank accession no. and range of amino acids included in construct**	**Protein name in this paper**	**Size (kDa)**	**Cloning strategy***	**Primer pairs**
Chitinophaga pinensis	ACU61621.1 429-552	*Cp*CBM92F	15.9	Cloned from gDNA into pET21a	5'-GGATCCATTGGTCAGACAGTGAC-3'<br>5'-CTCGAGCTGGTTGATTTTGAACTT-3'
Chitinophaga pinensis	ACU61949.1 788-912	*Cp*CBM92G	15.8	Cloned from gDNA into pET21a	5'-GGATCCGTAGGCAGCATTATCTAC-3'<br>5'-CTCGAGTGCAGCCGTCGTAGTA-3'
Draconibacterium mangrovi	WP_163322777.1 534-666	*Dm*CBM92A	16.9	Commercial synthesis	none
Aquimarina aggregata	WP_066313671.1 787-919	*Aa*CBM92A	17.2	Commercial synthesis	none
Euryarchaeota archaeon	MBS73237.1 119-254	*Ea*CBM92A	17.9	Commercial synthesis	none
Aquimarina aggregata	WP_066320065.1 456-596	*Aa*CBM92B	18.2	Commercial synthesis	none
Pyxidicoccus caerfyrddinensis	WP_164000589.1 27-170	*Pc*CBM92A	17.6	Commercial synthesis	none

*Commercial synthesis = gene was synthesised by ThermoFisher GeneArt, then transferred into plasmid pET21a by restriction digestion cloning.

Supplementary Table 3. Primers used in the mutagenesis of F1, and the site of each mutation. To generate the double and triple mutants, multiple rounds of mutagenic PCR were performed using the same primers.

**Mutation**	**Forward primer**	**Reverse primer**
W481A	5'-GTGATGCACCGCAGGCAgcGGAACTGTTTACCGTTG-3'	5'-CAACGGTAAACAGTTCCgcTGCCTGCGGTGCATCAC-3'
W523A	5'-GTAATCGTCCGGCAATTCAAGGTgcGGAAGCATTTGATTGGCTGG-3'	5'-CCAGCCAATCAAATGCTTCCgcACCTTGAATTGCCGGACGATTAC-3'
W565A	5'-GTCCGACCGCAAGCGGCgcGGAAGCCTTTGGTTATAG-3'	5'-CTATAACCAAAGGCTTCCgcGCCGCTTGCGGTCGGAC-3'

Supplementary Table 4. Conditions used for protein crystallisation.

**Protein-Ligand**	**Crystallisation condition**
*Cp*CBM92A	0.1 M Bis-Tris pH 5.5 with 10 % w/v PEG 3350, 400 mM NH_4SO_4, and 10 mM urea
*Cp*CBM92B	0.1 M citrate buffer pH 5.5 with 15 % w/v PEG 3350
*Cp*CBM92B-Glucose	0.1 M citrate buffer pH 5.5 with 15 % w/v PEG 3350
*Cp*CBM92B-Gentiobiose	0.1 M citrate buffer pH 5.5 with 12.5 % w/v PEG 3350
*Cp*CBM92B-Sophorose	0.1 M Bis-Tris pH 5.5 with 25 % w/v PEG 3350

Supplementary Table 5: Table of crystallographic statistics. Data in parentheses is for the highest resolution shell.

	*Cp*CBM92A	*Cp*CBM92B	*Cp*CBM92B-Glucose	*Cp*CBM92B-Gentiobose	*Cp*CBM92B-Sophorose
Data Collection					
Date	November 27, 2019	March 27, 2020	November 27, 2019	November 27, 2019	June 11, 2020
Source	BioMAX at MAXIV	BioMAX at MAXIV	BioMAX at MAXIV	BioMAX at MAXIV	BioMAX at MAXIV
Wavelength (Å)	0.991874	1.0332	0.991873	0.991873	0.976259
Space group	$P2_12_12_1$	P1	C121	C121	C121
Cell dimensions					
a, *b*, *c* (Å)	31.66, 53.71, 56.56	34.04, 59.06, 68.03	43.08, 111.97, 71.31	44.62, 111.41, 70.54	38.39, 113.05, 70.90
α, β, γ (°)	90.0, 90.0, 90.0	90.98, 92.55, 105.64	90.0, 100.32, 90.0	90.0, 98.58, 90.0	90.0, 98.95, 90.0
No. of measured reflections	166098 (7184)	247212 (22467)	209913 (14462)	185748 (12213)	120878 (3613)
No. of independent reflections	13501 (675)	69506 (6543)	31344 (2569)	27998 (2117)	18228 (911)
Resolution (Å)	38.95 - 1.40 (1.46 - 1.40)	33.97 - 1.56 (1.61 - 1.56)	39.64 - 1.77 (1.84 - 1.77)	29.56 - 1.85 (1.91 - 1.85)	43.99 - 1.68 (1.86 - 1.68)
Ellipsoidal resolution limit (Å)[1]	1.346 [a*]	-	-	-	2.667 [0.892 a* - 0.453 c*]
	1.312 [b*]	-	-	-	1.598 [b*]
	2.087 [c*]	-	-	-	1.792 [0.067 a* + 0.998 c*]
R_{merge}[2]	0.060 (0.71)	0.075 (1.13)	0.056 (0.91)	0.060 (0.40)	0.10 (0.81)
$CC_{1/2}$ (%)	99.8 (94.2)	99.6 (41.5)	99.9 (84.3)	99.9 (94.7)	99.7 (47.3)
$\langle I/\sigma(I) \rangle$	17.3 (3.0)	8.74 (1.04)	14.65 (1.37)	15.25 (2.42)	8.6 (1.2)
Completeness spherical (%)	68.8 (30.1)	95.35 (88.81)	97.03 (79.69)	95.66 (72.65)	54.0 (10.1)
Completeness ellipsoidal (%)	94.2 (70.1)	-	-	-	87.4 (47.3)
Redundancy	12.3 (10.6)	3.6 (3.4)	6.7 (5.6)	6.6 (5.8)	6.6 (4.0)
Refinement					
R_{work}/R_{free}	20.5/24.8	17.5/20.6	19.8/23.4	19.2/21.6	21.3/26.3
No. atoms					
Protein	947	3951	1963	1918	1974
Ligand/ions	0	32	80	52	28
Water	51	313	101	128	102
Average B-factors					
Protein	16.37	29.43	41.53	41.53	29.60
Ligand/ions	-	31.41	51.07	46.81	33.11
Water	21.05	38.59	44.91	44.91	30.84
RMSD from ideal geometry[3]					
Bond length (Å)	0.006	0.006	0.006	0.006	0.007
Bond angles (°)	0.88	0.81	0.72	0.76	0.89
PDB accession	7ZOI	7ZOH	7ZON	7ZOO	7ZOP

[1] Brackets represent the direction along the reciprocal lattice. [2] $R_{merge} = \sum_{hkl}\sum_i |I_i(hkl) - \langle I(hkl)\rangle| / \sum_{hkl}\sum_i I_i(hkl)$, wherein $I_i(hkl)$ is the intensity of the *i*th measurement of reflection *hkl*, and $\langle I(hkl)\rangle$ is the mean value of $I_i(hkl)$ for all the *i* measurements. [3] Root mean square deviations from ideal geometry values, as per Engh & Huber, Acta Crystallogr. Sect. A. **47**, 392–400 (1991).
