# Supplementary material for: Structural and biochemical analysis of family 92 carbohydrate-binding modules uncovers multivalent binding to β-glucans
Source: Nat Commun. 2024 Apr 23;15:3429. doi: 10.1038/s41467-024-47584-y (PMC11039641; doi:10.1038/s41467-024-47584-y)
Supplement: Supplementary file 2 — Reporting Summary [file 41467_2024_47584_MOESM2_ESM.pdf]

nature portfolio

Corresponding author(s): Lauren McKee

Last updated by author(s): Jan 25, 2024

Reporting Summary

Nature Portfolio wishes to improve the reproducibility of the work that we publish. This form provides structure for consistency and transparency in reporting. For further information on Nature Portfolio policies, see our Editorial Policies and the Editorial Policy Checklist.

Statistics

For all statistical analyses, confirm that the following items are present in the figure legend, table legend, main text, or Methods section.

n/a	Confirmed	
☐	☒	The exact sample size (n) for each experimental group/condition, given as a discrete number and unit of measurement
☐	☒	A statement on whether measurements were taken from distinct samples or whether the same sample was measured repeatedly
☒	☐	The statistical test(s) used AND whether they are one- or two-sided<br>*Only common tests should be described solely by name; describe more complex techniques in the Methods section.*
☒	☐	A description of all covariates tested
☒	☐	A description of any assumptions or corrections, such as tests of normality and adjustment for multiple comparisons
☐	☒	A full description of the statistical parameters including central tendency (e.g. means) or other basic estimates (e.g. regression coefficient) AND variation (e.g. standard deviation) or associated estimates of uncertainty (e.g. confidence intervals)
☒	☐	For null hypothesis testing, the test statistic (e.g. F, t, r) with confidence intervals, effect sizes, degrees of freedom and P value noted<br>*Give P values as exact values whenever suitable.*
☒	☐	For Bayesian analysis, information on the choice of priors and Markov chain Monte Carlo settings
☒	☐	For hierarchical and complex designs, identification of the appropriate level for tests and full reporting of outcomes
☒	☐	Estimates of effect sizes (e.g. Cohen's d, Pearson's r), indicating how they were calculated

Our web collection on statistics for biologists contains articles on many of the points above.

Software and code

Policy information about availability of computer code

Data collection	The ThermoFisher GeneArt online tool was used to design gene constructs that were synthesised. Snapgene version 5.3 was used for design of primers for genes cloned in-house from genomic DNA. Photographs of SDS-PAGE gels were taken using a mobile phone camera and transferred into Adobe Illustrator 2022 for annotation. The software CFX Manager was used to observe and export data from DSF experiments. The software Origin 2019 (OriginLab) was used to observe and export ITC data. Raw data from BLI experiments were exported using the Octet software and visualised using MatLab version E2021a.
Data analysis	The following software and online tools were used in bioinformatic analyses: NCBI BLAST, NCBI Taxonomy, Clustal Omega and Clustal Muscle from the European Molecular Biology Laboratory (EMBL) (https://www.ebi.ac.uk/Tools/msa); GENEIOUS Prime® 2022.0.1; IQtree multicore version 1.6.12 for Linux 64-bit built Aug 15 2019; Interactive Tree Of Life (iTOL) version 6.5.<br>The following were used for analysis of data related to protein structure determination: STARANISO - Open source online server for processing of anisotropic macromolecular crystallography datasets. https://staraniso.globalphasing.org/cgi-bin/staraniso.cgi; XDS - version 20210205 - Open source software for data processing of macromolecular datasets. https://xds.mr.mpg.de/; Phenix - version 1.19.2-4158 - Open source software for structure determination and model building of macromolecular crystallography datasets. https://phenix-online.org/; CCP4 online - Open source online server for structure determination and model building of macromolecular crystallography datasets. https://ccp4online.ccp4.ac.uk/ccp4online/; ARP/wARP - Open source online server for structure determination and model building of macromolecular crystallography datasets. https://arpwarp.embl-hamburg.de/.<br>All figures were assembled and annotated in Adobe Illustrator 2022. Tiff files were generated from these images using Adobe Photoshop 2022.

For manuscripts utilizing custom algorithms or software that are central to the research but not yet described in published literature, software must be made available to editors and reviewers. We strongly encourage code deposition in a community repository (e.g. GitHub). See the Nature Portfolio guidelines for submitting code & software for further information.

Data

Policy information about availability of data

All manuscripts must include a data availability statement. This statement should provide the following information, where applicable:

- Accession codes, unique identifiers, or web links for publicly available datasets
- A description of any restrictions on data availability
- For clinical datasets or third party data, please ensure that the statement adheres to our policy

Accession codes for all proteins used in bioinformatic or biochemical analysis are provided in Supplementary Tables S1 and S2. Coordinates and structure factors for CpCBM92A, CpCBM92B, and CpCBM92B in complex with glucose, gentiobiose, and sophorose have been deposited in the RCSB Protein Data Bank under accession codes 7ZOI, 7ZOH, 7ZON, 7ZOO, and 7ZOP, respectively. All other data generated or analysed during this study are included in this published article (and its supplementary information files).

Research involving human participants, their data, or biological material

Policy information about studies with human participants or human data. See also policy information about sex, gender (identity/presentation), and sexual orientation and race, ethnicity and racism.

Reporting on sex and gender	N/A
Reporting on race, ethnicity, or other socially relevant groupings	N/A
Population characteristics	N/A
Recruitment	N/A
Ethics oversight	N/A

Note that full information on the approval of the study protocol must also be provided in the manuscript.

Field-specific reporting

Please select the one below that is the best fit for your research. If you are not sure, read the appropriate sections before making your selection.

- [x] Life sciences
- [ ] Behavioural & social sciences
- [ ] Ecological, evolutionary & environmental sciences

For a reference copy of the document with all sections, see nature.com/documents/nr-reporting-summary-flat.pdf

Life sciences study design

All studies must disclose on these points even when the disclosure is negative.

Sample size	Twelve CBM proteins were selected for characterisation as this represented close to 10 % of the total number of proteins identified in our phylogeny. Many CBM families existing on the database have a smaller number of characterised examples but we wanted to have confidence when stating the specificity of the family. The sample size is sufficient for a broad survey of the protein family because we selected diverse sequences from around the tree, and we selected domains appended to a representative survey ot catalytic domains. Nonetheless, we do not state that our observations will necessarily apply to every other yet-to-be-characterised CBM92 protein.
Data exclusions	No data were excluded from experimental datasets presented in the manuscript. Two additional genes were cloned but the proteins could not be stably produced in recombinant form in E.coli, so they were not characterised and are not included in the paper. For proteins that were studied, certain experiments were not feasible (e.g. NativePAGE analysis could not be performed due to a high protein isoelectric point).
Replication	Biochemical assays performed in triplicate as standard (e.g. DSF).<br>Qualitative polysaccharide binding assays: proteins were assayed for binding to a series of polysaccharides. The assay for each protein was performed three times, and the same results were obtained each time. One representative SDS-PAGE image is provided for each protein.<br>Quantitative polysaccharide binding assays: absorbance for each sample was measured three times, and the whole experiment was performed twice for each protein (using different production batches of protein).<br>All replicate experiments were successful, and none were excluded from analysis.
Randomization	Randomisation is not relevant to this study - all proteins were subjected to the same analysis in binding assays. Glycans used in the structural biology, BLI, QDI, and ITC experiments were systematically chosen to represent ligands identified in the pull-down assays.
Blinding	Blinding and sample grouping are not relevant to this study. All proteins were analysed for binding in the same way.

Reporting for specific materials, systems and methods

We require information from authors about some types of materials, experimental systems and methods used in many studies. Here, indicate whether each material, system or method listed is relevant to your study. If you are not sure if a list item applies to your research, read the appropriate section before selecting a response.

Materials & experimental systems

n/a	Involved in the study
☒	☐ Antibodies
☒	☐ Eukaryotic cell lines
☒	☐ Palaeontology and archaeology
☒	☐ Animals and other organisms
☒	☐ Clinical data
☒	☐ Dual use research of concern
☒	☐ Plants

Methods

n/a	Involved in the study
☒	☐ ChIP-seq
☒	☐ Flow cytometry
☒	☐ MRI-based neuroimaging

Plants

Seed stocks

Report on the source of all seed stocks or other plant material used. If applicable, state the seed stock centre and catalogue number. If plant specimens were collected from the field, describe the collection location, date and sampling procedures.

Novel plant genotypes

Describe the methods by which all novel plant genotypes were produced. This includes those generated by transgenic approaches, gene editing, chemical/radiation-based mutagenesis and hybridization. For transgenic lines, describe the transformation method, the number of independent lines analyzed and the generation upon which experiments were performed. For gene-edited lines, describe the editor used, the endogenous sequence targeted for editing, the targeting guide RNA sequence (if applicable) and how the editor was applied.

Authentication

Describe any authentication procedures for each seed stock used or novel genotype generated. Describe any experiments used to assess the effect of a mutation and, where applicable, how potential secondary effects (e.g. second site T-DNA insertions, mosiacism, off-target gene editing) were examined.
